# *Peumus boldus*: Revealing the Pharmaceutical Potential of the Chilean Forest

**DOI:** 10.1002/cbdv.71690

**Published:** 2026-10-27

**Authors:** Marta Rodríguez‐Ceballos, Valentina Jara, Sofía Díaz‐Cornejo, Valentina Morales, Rómulo Santelices‐Moya, Miguel Aguilera, Cristian Atala, María Carolina Otero, Felipe Gordillo‐Fuenzalida

**Affiliations:** ^1^ Facultad De Ciencias Agrarias y Forestales Universidad Católica del Maule, Centro de Biotecnología de Los Recursos Naturales (CENBio) Talca Chile; ^2^ Facultad De Ciencias Agrarias y Forestales Universidad Católica del Maule, Centro de Desarrollo para El Secano Interior Talca Chile; ^3^ Laboratorio De Anatomía y Ecología Funcional De Plantas (AEF) Facultad De Ciencias Instituto De Biología Campus Curauma Pontificia Universidad Católica De Valparaíso Valparaíso Chile; ^4^ Escuela De Química y Farmacia, Facultad De Medicina Universidad Andres Bello Santiago Chile

**Keywords:** alkaloids, *Peumus boldus*, phenolic compounds

## Abstract

*Peumus boldus* Molina (boldo) is a slow‐growing tree endemic to central Chile and the only South American representative of the Monimiaceae family. Adapted to Mediterranean climatic conditions, it possesses sclerophyllous leaves and deep root systems that allow tolerance to drought and high solar radiation. Ecologically, plays an important role in the Chilean sclerophyllous forest and contributes to post‐fire regeneration processes. The species is widely recognized for its medicinal value due to its rich phytochemical composition, particularly the presence of boldine, an aporphine alkaloid associated with several pharmacological properties. In addition, contains phenolic compounds and terpenoid rich volatile fractions that contribute to its biological activity. Studies have demonstrated antioxidant, anti‐inflammatory, and antimicrobial properties of its extracts and isolated compounds. These effects are mainly associated with free radical scavenging, modulation of inflammatory pathways, and inhibitory activity against bacterial and fungal strains. Other potential activities, including hepatoprotective and neuroprotective effects, have also been reported. This review also integrates current knowledge on the molecular mechanisms underlying these effects, highlighting the modulation of oxidative stress and inflammatory signaling pathways. In addition, extraction technologies, safety considerations, and existing knowledge gaps are critically discussed. Future pharmaceutical development should focus on isolating beneficial compounds like boldine to minimise systemic risks. At the same time, public health guidelines should emphasize avoiding self‐medication with concentrated extracts and the strict monitoring of patients with pre‐existing liver conditions or who are pregnant.

## Graphical abstract

*P. boldus* emerge as a promising yet underexplored source of bioactive compounds. Current evidence highlights antioxidant, anti‐inflammatory, antimicrobial, and cytoprotective activities associated with their unique phytochemical profile. This review integrates chemical composition, biological properties, proposed molecular mechanisms, and safety considerations, providing a comprehensive perspective on their therapeutic and biotechnological potential.

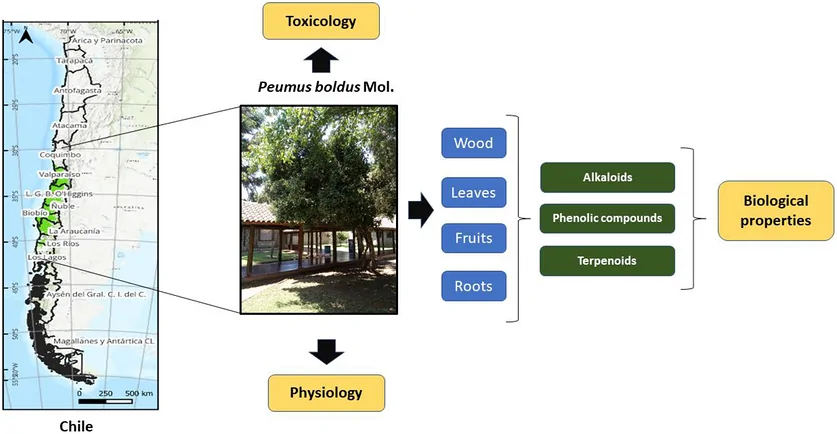

## 1 Introduction

*Peumus boldus* Mol., known in Chile as boldo, is a small evergreen tree that thrives exclusively in the humid ecosystems of the Mediterranean climate region in Chile [1]. Botanically described for the first time by Abate Juan Ignacio Molina two centuries ago, this tree belongs to the Monimiaceae family, a pantropical family composed of 26 genera and around 250 species, which includes trees, shrubs, and evergreen vines [1]. *P. boldus* is a dioecious endemic plant and represents a characteristic component of the sclerophyllous forest [2]. Mature boldo trees typically reach heights of between 6 and 12 m, although some specimens can grow up to 20 m. This medium‐sized tree presents a slow rate of growth, reaching adult size in several decades [3]. Once the trunk is cut, the tree can appear as a shrub with several main stems. It has simple, aromatic, ovate‐elliptic leaves, with a dark green, densely glandular upper side, and a light grayish‐green underside [4].

*P. boldus* is highly appreciated for its health benefits; in fact, it is one of the most important Chilean medicinal plant known, traditionally used in native ancestral medicine [5, 6, 7]. Furthermore, the medicinal use of *P. boldus* leaves has a long history spanning approximately 14 500 years. Archaeological evidence from the Monte Verde settlement has recovered chewed pellets containing a mixture of three to four species of marine algae along with *P. boldus* plant material [8]. These findings constitute valuable evidence of the traditional and ancestral use of this species in therapeutic contexts, and provide relevant information for interdisciplinary studies in ethnobotany and pharmacological archaeology.

Due to these characteristics, *P. boldus* is one of the most economically and environmentally valuable endemic forest species of Chile [3]. Its traditional use, as well as its widespread use in international pharmacopoeias, have crossed borders, so that since the late nineteenth century leaves exports began with exponential growth in the last two decades [3]. For the year 2025, export of boldo (i.e., bark, flowers, and leaves) reached a value of US$ FOB of 6.111.147, mainly to Argentina, Brazil, Italy, France, and Germany, among other countries [9]. However, other products derived from this specie include essential oils (EOs), encapsulated powders, herbal teas, and fresh fruits [10, 11, 12, 13]. At least two factors have contributed to the recent resurgence of interest in this specie; the accumulation of extensive knowledge based on its chemical composition and its widespread cultural perception as an effective medicinal plant for the treatment of health disorders [6, 7, 13, 14].

Leaves of *P. boldus* have been used in folk medicine to treat different affections such as headache, earache, rheumatism, dropsy, dyspepsia, liver and gallbladder affections, stomach ache, menstrual pain and urinary tract inflammation [5, 6, 7, 15]. These effects are attributed to the broad spectrum of pharmacological activities exhibited by *P. boldus* extracts, including cholagogue, antioxidant, anti‐inflammatory, hepatoprotective, antimicrobial, and diuretic properties, among others, which contribute to the prevention and management of diseases associated with oxidative stress [6, 7]. Scientific research on *P. boldus* has focused mainly on its leaves, which exhibit mainly antioxidant activity, owing to their high phenolic compound content and the presence of other bioactive compounds, such as alkaloids [13, 14]. However, leaves also contained EOs, which have been found to contain antimicrobial, insecticidal, and herbicidal properties probably due to their terpenoid content [16]. The content of these secondary metabolites in *P. boldus* could vary considerably depending on the tissue of the plant, extraction procedure, analytical method used, and other environmental parameters [13, 14]. The presence of several metabolites in the plant highlights the importance of continuously updating our knowledge of chemical diversity and pharmacological potential of this endemic plant. In the last decade, there has been an exponential increase in the number of articles featuring the potential uses of *P. boldus* and its constituents and future research may further unravel its chemical complexity. In this review, we compilated the biological properties of this specie of high scientific and conservation value, representing a paradigmatic model of adaptation to the Mediterranean climate. Its study offers valuable evolutionary, physiological, and pharmacological implications, including toxicity, fundamental for deepening our understanding of South America's floristic diversity and promoting effective conservation and sustainable management strategies.

## 2 Material and Methods

A literature review was conducted through the collection and analysis of information from scientific articles and clinical studies retrieved from indexed databases. The search was performed using the electronic platforms “Web of Science”, “Scopus”, and “NCBI/PubMed”. The literature search was conducted between December and March of 2025.

The following Boolean search expression was applied: (“*Peumus boldus*”) AND (“physiology” OR “antioxidant” OR (“antimicrobial” OR “antiviral” OR “antibacterial” OR “antifungal”) OR “anti‐inflammatory” OR “metabolites” OR “toxicity”).

Studies published between 2015 and 2025 were considered for inclusion. The eligibility criteria included: (i) experimental studies and clinical investigations related to *P. boldus* and its physiological, pharmacological, antimicrobial, antioxidant, anti‐inflammatory, metabolite, or toxicity‐related properties; (ii) full‐text articles available in peer‐reviewed journals; and (iii) studies published in English.

The exclusion criteria were: (i) duplicate records; (ii) articles not directly related to the objectives of this review; (iii) conference abstracts, editorials, and non‐peer‐reviewed publications; and (iv) studies with insufficient methodological information.

The selection process was conducted through title and abstract screening followed by full‐text assessment according to the eligibility criteria. A total of 187 records were initially identified across all databases. After removing duplicate records and excluding studies not relevant to the review objectives, 85 studies were retained for final analysis. The study selection process was summarized using a PRISMA flow diagram (Figure 1). Additional references were incorporated during manuscript revision to address specific mechanistic, phytochemical, and toxicological aspects suggested by reviewers. These references were not considered part of the systematic evidence synthesis and were used exclusively to provide contextual and mechanistic support.

**FIGURE 1:**
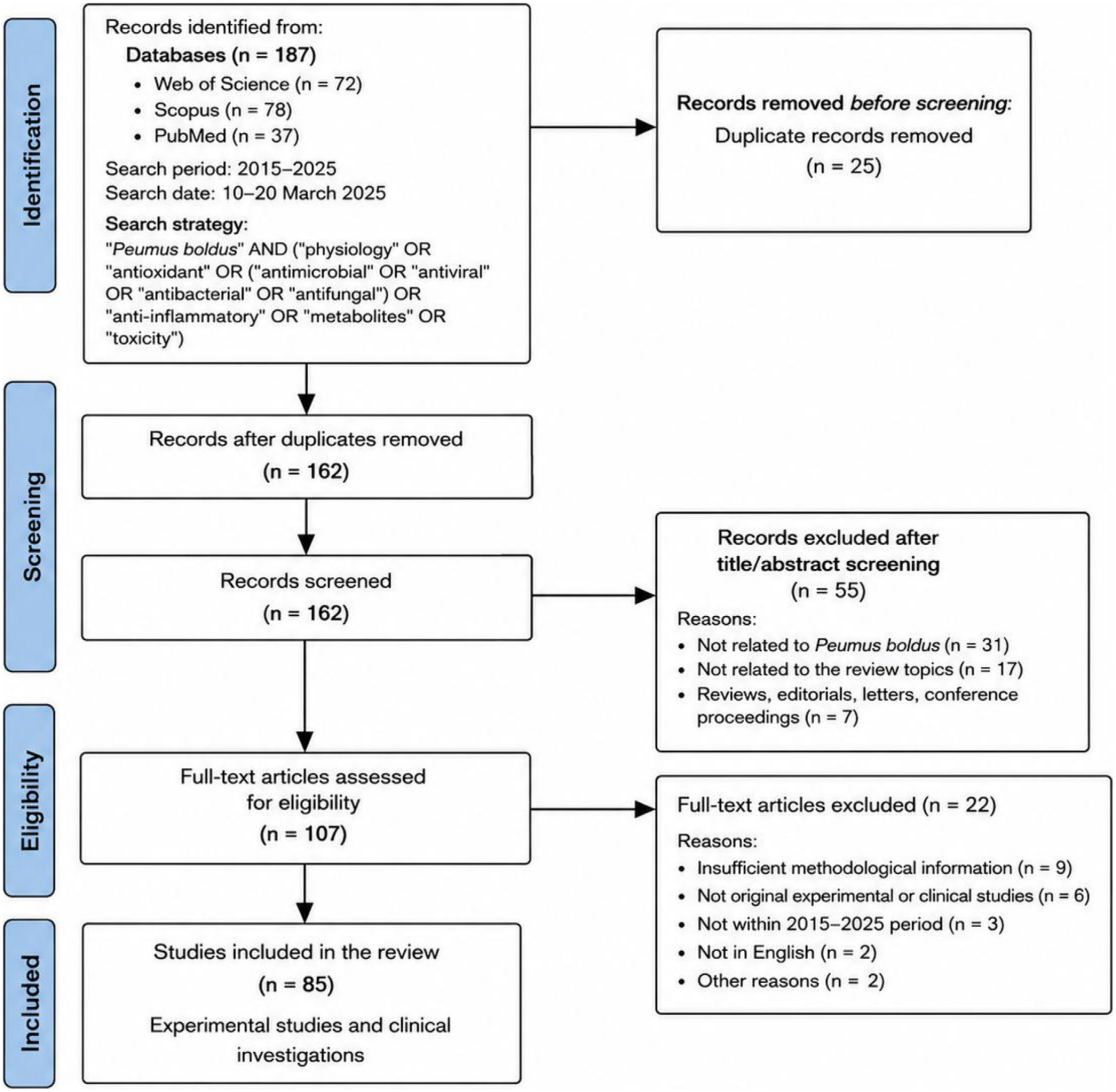
PRISMA flow diagram illustrating the literature search and study selection process. A total of 187 records were identified through searches in Web of Science, Scopus, and NCBI/PubMed using predefined search terms. After the removal of duplicate and nonrelevant records, studies were screened according to the eligibility criteria, resulting in the inclusion of 85 studies published between 2015 and 2025 for qualitative synthesis.

## 3 Physiological Aspects of *P. boldus* Mol

*P. boldus*, called folo in Mapudungun (the language of the Mapuche people), is remarkably the only representative of the Monimiaceae family in South America [1]. *P. boldus* is a tree endemic to Chile, growing between 33° and 39° south latitude, this is primarily spanning from the Coquimbo to the Los Lagos regions (Figure 2). It grows primarily on low, sunny slopes of both mountain ranges, as well as in the central valley. Regarding its altitudinal range, this specie is distributed between 0 and 1500 meters above sea level [1]. This latitudinal distribution closely coincides with the Chilean Mediterranean climate, characterized by wet winters and dry summers [17]. The area with a significant presence of *P. boldus*, considering the first two dominance strata in native forests, reaches approximately 302.329 ha. The highest concentration of this tree is located in the Maule region (23% of the total area detected), followed by the Metropolitan of Santiago (21%) and Valparaíso regions (19%) [18]. These demonstrate the regional importance of the specie and its contribution both to the structure of the Chilean sclerophyllous forest and to the provision of ecosystem services in the temperate Mediterranean environment.

**FIGURE 2:**
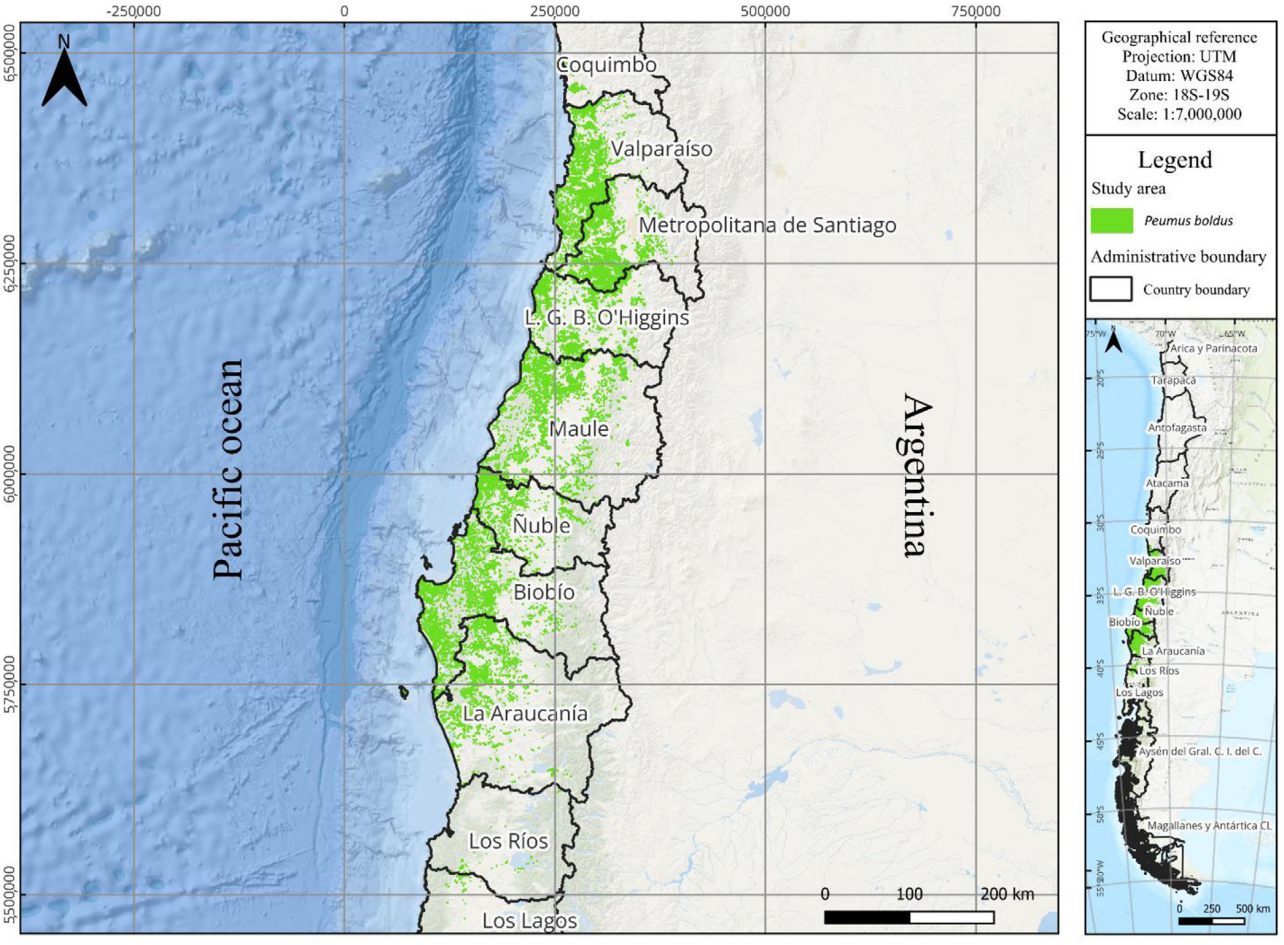
Natural distribution of *P. boldus* Mol. At the national level, the area covered by *P. boldus* (considering its presence in the first two dominance strata) reaches 302.329 ha. The Maule Region concentrates the largest proportion of the resource (23%), followed by the Metropolitan of Santiago (21%) and Valparaíso (19%) Regions (IDE_MINAGRI 2025).

The specie exhibits dioecious characteristics, with functionally separate male and female individuals. Flowering occurs between September and December, and pollination is entomophilous, carried out primarily by hymenopterans. It produces its aromatic edible fruits between December and February [5, 19]. The fruits are fleshy, juicy, green, ovoid, sweet and with a pleasant aroma, containing a single seed and clustered in groups of 2 to 5. Length is between 6 and 8 mm, with yellow to green color, and remains attached to the peduncle when it is mature [10, 19] (Figure 3). Fruit dispersal is zoochorous, facilitated by birds and mammals that consume the fruits and transport the seeds to new locations [20]. Under optimal conditions, *P. boldus* can reach heights exceeding 10 meters and live for over 100 years, demonstrating remarkable longevity within Mediterranean ecosystems.

**FIGURE 3:**
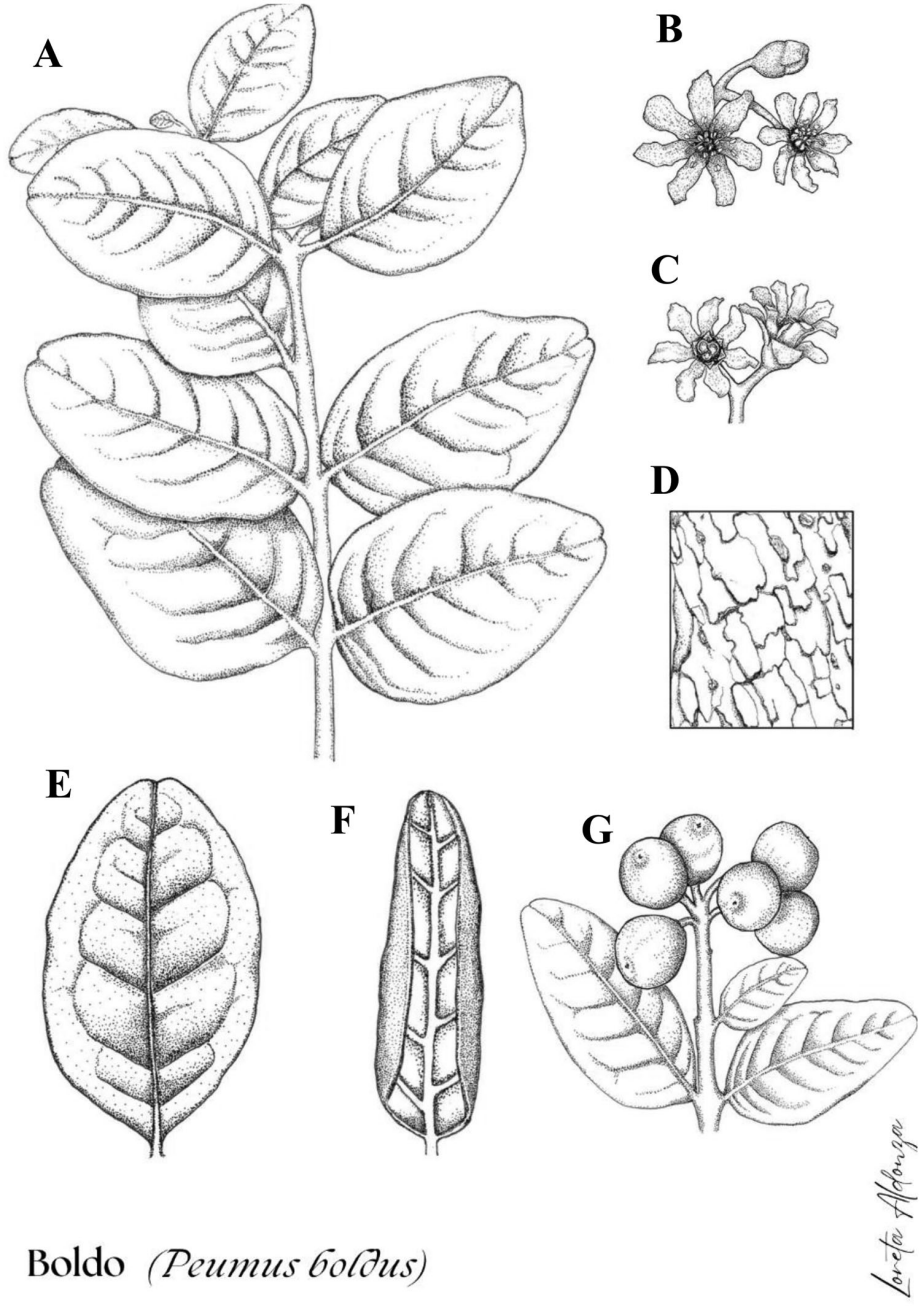
Some parts of the *P. boldus* plant. (A) Branch with leaves, (B) male flower, (C) female flower, (D) tree bark, (E) obverse tree leaf, (F) reverse tree leaf, (G) leaves and fruits. Original illustration created specifically for this manuscript by Loreta Aldonza and used with permission.

This species seems to be relatively basal in the Monimiaceae phylogeny, probably evolving from an African ancestor [21]. It has developed remarkable adaptations that allow it to thrive in the Chilean Mediterranean ecosystem, becoming a distinctive element of the sclerophyllous forest and a paradigmatic example of evolutionary specialization to specific environmental conditions; it has a greater tolerance than other species with which it shares its habitat in periods of water stress, which allows it to occupy areas with lower water availability [22]. It has a slightly higher specific leaf area compared to *Quillaja saponaria*, (Quillajaceae), but similar to *Colliguaja odorifera* (Euphorbiaceae) [23]. *P. boldus* is a slow‐growing specie [24], but management practice such as selective pruning can increase biomass accumulation and water status [25]. It can naturally associate with arbuscular mycorrhizal fungi (AMF) such as *Funneliformis* spp., which can help regeneration after fires [26, 27].

The life cycle of *P. boldus* begins with seed germination, which depends on breaking dormancy. Several studies have documented the difficulties associated with this process. Recently, Santelices‐Moya et al., 2020 demonstrated that removing the epicarp and mesocarp from seeds harvested at the beginning of their maturation significantly increases the germination rate, reaching 51,4%. This value represents the highest percentage reported for this species under controlled conditions, achieving this effect without the application of gibberellic acid as a germination promoter. The seedlings develop foliaceous cotyledons and exhibit slow initial growth, displaying a monopodial development pattern that can result in shrubby or tree‐like forms depending on site conditions [28].

*P. boldus* has developed various physiological strategies to cope with the conditions of the Mediterranean climate, characterized by prolonged summer drought and high solar radiation. In the northern provenances of the specie, an adaptive response has been observed that includes a reduction in leaf size, an increase in the allocation of resources to root biomass, and delayed germination, compared to southern provenances [29]. These adaptations promote water use efficiency and resistance to the water restriction typical of its habitat, optimizing water absorption through deep roots and decreasing water loss through foliar transpiration under environmental stress conditions. Morphological adaptations developed by xerophytic species such as *P. boldus* include sclerophyllous evergreen leaves and notable thickness, a thick cuticle, sunken stomata of reduced dimensions, and microscopic star‐shaped trichomes that regulate water loss and provide protection against excessive ultraviolet radiation [30, 31].

Although specific information on the vascular system of *P. boldus* is lacking, it is estimated, based on its habitat characteristics, that this specie could exhibits typical xeromorphic adaptations. These include xylem vessels with relatively small diameters and thickened walls, which reduces the risk of cavitation during periods of severe water deficit [32]. There is also no specific information on the photosynthetic metabolism of *P. boldus*, although it is estimated that, like most plants, it follows the C3 pathway. This specie may exhibit adaptations that optimize photosynthetic efficiency under water stress conditions like increased water‐use efficiency [23].

From a phylogenetic perspective, *P. boldus* is placed within the family Monimiaceae and the order Laurales within the magnoliid clade. *P. boldus* constitutes a monospecific genus, making *P. boldus* a phylogenetically isolated lineage within its family. The characteristic phytochemical profile of *P. boldus*, particularly the exceptionally high concentrations of boldine alkaloid in bark, around 7% of the total dry weight, highlights its uniqueness within the order Laurales [13, 14]. Although this alkaloid is present in related species, it only reaches commercially significant levels in *P. boldus*, which is interpreted as a biochemical specialization with evolutionary relevance. This high accumulation not only contributes to the chemical identity of the specie but is also associated with its pharmacological properties, reinforcing its importance from both an ecological and biotechnological perspective.

## 4 Comparison of Extraction Technologies in *P. boldus*

The phytochemical profile of *P. boldus* is strongly influenced by extraction conditions, with both extraction yield and molecular diversity depending on the methodology employed, solvent polarity, and matrix–solvent interactions. A comparative analysis of the literature reveals a consistent extraction profile whereby conventional aqueous methods, such as infusions and decoctions, preferentially recover hydrophilic low‐molecular‐weight phenolic compounds, particularly hydroxycinnamic and hydroxybenzoic acid derivatives, together with selected flavonoids including glycosylated and highly hydroxylated compounds, whereas the extraction efficiency of the major aporphine alkaloid, boldine, remains comparatively low [33]. This limitation is largely attributed to the intermediate polarity and aromatic structure of boldine, which reduce its solubilization in purely aqueous systems and often require amphiphilic solvents or elevated temperatures to facilitate its release from plant matrix interactions. Consequently, hydroalcoholic mixtures (50%–70% ethanol or 100 % methanol) generally provide superior extraction efficiency by promoting matrix swelling and improving solubility across a wider polarity spectrum, thereby enabling a more balanced recovery of both phenolic and alkaloid fractions [34]. Matrix swelling induced by solvent penetration can increase cell wall permeability and enhance mass transfer, thereby improving the release of intracellular metabolites during extraction [35].

In recent years, this paradigm has evolved toward greener extraction strategies within the framework of advanced extraction technologies (AETs). Microwave‐assisted extraction (MAE) and ultrasound‐assisted extraction (UAE) have gained considerable attention due to their ability to substantially reduce extraction time and solvent consumption through dielectric heating and acoustic cavitation, respectively. However, under prolonged irradiation or excessive processing conditions, MAE may promote thermal degradation of sensitive polyhydroxylated flavonoids, whereas UAE generally favors the preservation of thermolabile compounds because of its lower operating temperatures and milder energy input [36, 37]. In parallel, supercritical fluid extraction (SFE‐CO_2_) has emerged as a highly selective technique for the recovery of volatile monoterpenes, including ascaridole, eucalyptol, *O*‐cymene, and fenchone [38, 39]. Nevertheless, the extraction efficiency of moderately polar compounds such as the boldine is restricted by the inherently low polarity of supercritical CO_2_. This limitation can be partially overcome by incorporating polar co‐solvents, particularly ethanol, which modulate solvent polarity and broaden the applicability of SFE toward alkaloid‐enriched and solvent‐reduced extracts [39].

Beyond extraction methodology, recent phytochemical profiling studies have challenged the assumption of a homogeneous metabolite distribution within *P. boldus*. Available evidence indicates marked tissue‐specific metabolite compartmentalization across different plant organs. Boldine has been reported at higher abundance in bark tissues, whereas leaves exhibit a more diverse alkaloid composition characterized by metabolites such as *N*‐methyl‐laurotetanine, laurotetanine, and coclaurine. In contrast, woody tissues and roots appear to preferentially accumulate laurolitsine, while fruits display a distinct mixed phytochemical profile in which alkaloids, including higenamine and isocorydine, coexist with phenolic compounds such as chlorogenic acid, rutin, and catechin derivatives [33]. Collectively, these findings indicate that the chemical signature of *P. boldus* is determined not only by extraction conditions but also by organ‐specific biosynthetic compartmentalization.

## 5 Chemical Composition of *P. boldus*

*P. boldus* leaves contain a complex mixture of secondary metabolites, primarily including alkaloids, phenolic compounds (flavonoids and phenolic acids), and EOs, which collectively account for the biological activities reported in vitro and in vivo. Leaf EOs are characterized by a diverse terpenoid profile comprising monoterpenes, oxygenated monoterpenes, sesquiterpenes, oxygenated sesquiterpenes, and non‐terpenoid compounds such as fatty acids, hydrocarbons, aldehydes, ketones, and aromatic constituents. In addition to boldine, these fractions also include isoquinoline and aporphine alkaloids, whose identification depends strongly on extraction strategy and analytical platform [16]. Furthermore, phenolic acids, flavonoids, and terpenoids complement these activities with the species’ antioxidant, antimicrobial, and anti‐inflammatory potential. A summary of the chemical structures of metabolites found in extracts presenting biological activity is presented in Figure 4.

**FIGURE 4:**
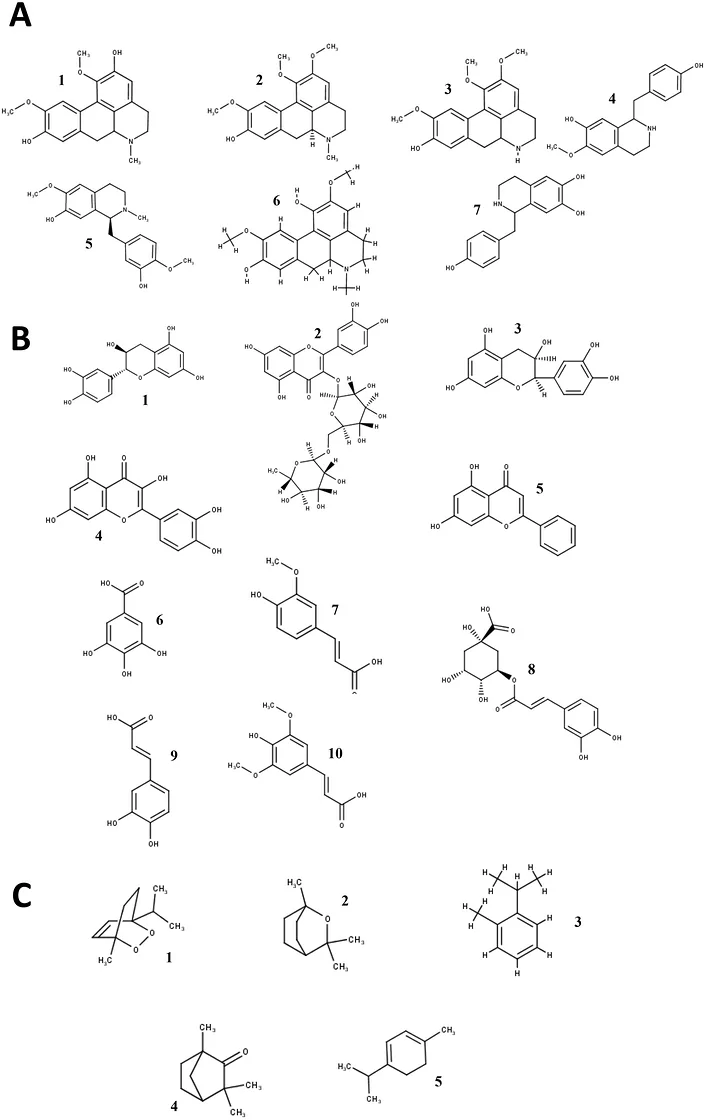
Chemical structure of metabolites found in *P. boldus* fractions with biological activity. (A) **Alcaloids**. 1: boldine; 2: *N*‐methyl‐laurotetanine; 3: laurotetanine; 4: coclaurine; 5: reticuline; 6: isoboldine; 7: higenamine. (B) **Phenolic compounds**. 1: catechin; 2: rutin; 3: epicatechin; 4: quercetin; 5: chrysin; 6: gallic acid; 7: ferulic acid; 8: chlorogenic acid; 9: caffeic acid; 10: sinapic acid. (C) **Terpenes**. 1: ascaridole; eucalyptol; *O*‐cymene; fenchone; α‐terpinene.

### 5.1 Essential Oil Composition and Analytical Variability

Gas chromatography–mass spectrometry (GC‐MS) analysis of hydrodistilled leaf EOs has consistently revealed a complex mixture of volatile compounds. Madrid et al., identified *O*‐cymene, β‐phellandrene, eucalyptol, fenchone, pinocarvone, terpinen‐4‐ol, terpineol, myrtenal, ascaridole, isoascaridole, methyleugenol, caryophyllene, bicyclogermacrene, spathulenol, globulol, and germacratrien‐1‐ol, with ascaridole (49.95%), *O*‐cymene (13.45%), and eucalyptol (9.71%) as dominant constituents [40]. In contrast, Lopes et al., using a comparable extraction and analytical approach, reported a broader monoterpene profile including α‐thujene, α‐pinene, sabinene, β‐pinene, α‐phellandrene, limonene, linalool, α‐terpinene, γ‐terpinene, and bornyl acetate, with ascaridole (35.24%), eucalyptol (29.27%), α‐terpinene (18.16%), and isoascaridole (3.59%) as major constituents [41]. These differences highlight substantial chemical plasticity influenced by environmental conditions, plant origin, and extraction parameters.

### 5.2 Alkaloid and Metabolite Profiling by Complementary Analytical Platforms

Beyond volatile fractions, multiple analytical strategies have expanded the known alkaloid diversity of *P. boldus*. A combination of spectral database mining and carbon‐13 nuclear magnetic resonance (^13^C NMR)‐based approaches has identified key alkaloids such as isocorydine, rogersine, boldine, reticuline, coclaurine, laurotetanine, *N*‐methylcoclaurine, and noisocorydine, alongside monoterpenes such as *p*‐cymene, eucalyptol, and α‐terpinene [42].

Ultrasound‐assisted extraction followed by high‐performance liquid chromatography coupled with ultraviolet detection and mass spectrometry (HPLC‐UV‐MS) and proton nuclear magnetic resonance (^1^H NMR) has revealed a predominant accumulation of phenolic compounds, particularly rutin, catechin, and epicatechin, with catechin often being the most abundant flavonoid [43]. Complementary, high‐performance liquid chromatography coupled with heated electrospray ionization tandem mass spectrometry (HPLC‐HESI‐MS/MS) analyses have identified additional polyphenols, including procyanidin C1, luteolin glycosides, cyanidin derivatives, and various flavonoid glycosides [44].

^1^H NMR profiling of aqueous extracts has further revealed a complex mixture of primary metabolites, including amino acids (alanine, asparagine, glutamine, phenylalanine, proline, tyrosine, valine), carbohydrates (glucose, fructose, sucrose), and organic acids (acetic, citric, formic, malic, succinic, malonic acids), supporting the complexity of crude herbal preparations beyond secondary metabolism. High‐resolution mass spectrometry (HRMS‐ESI) has confirmed a broader alkaloid repertoire, including coclaurine, *N*‐methylcoclaurine, laurolitsine, norreticuline, boldine, isoboldine, laurotetanine, reticuline, *N*‐methyl‐laurotetanine, isocoridine, and glaucine, along with phenolic derivatives such as luteolin and kaempferol glycosides and caffeoylquinic acid derivatives [45].

### 5.3 Aqueous Extracts, Standardization Approaches, and Phenolic Diversity

Aqueous extracts analyzed by high‐performance liquid chromatography coupled with a diode array detector (HPLC‐DAD) consistently revealed a mixture of boldine, gallic acid, rutin, and other phenolic acids [46]. Quantitative analyses further showed substantial levels of chlorogenic acid equivalents and anthocyanins, with rutin and kaempferol derivatives as dominant flavonoids, and cyanidin and peonidin as major anthocyanidins [47]. Similar profiles have been reported by da Silva et al., confirming the reproducibility of core phenolic signatures across aqueous preparations [48].

Extraction using natural deep eutectic solvents (NADES) has further improved recovery efficiency of alkaloids and phenolics. Proline–oxalic acid (1:1) systems with 20% water have been particularly effective in enriching boldine and total phenolic content. Liquid chromatography coupled with mass spectrometry (LC‐MS‐based) analyses confirm the presence of coclaurine, *N*‐methylcoclaurine, laurolitsine, isoboldine, boldine, reticuline, isocorydine, laurotetanine, and *N*‐methyl‐laurotetanine across different ionization platforms [12].

Morpho‐anatomical authentication combined with Soxhlet extraction and HPLC quantification of boldine has provided a methodological framework for quality control and standardization of *P. boldus*‐based preparations, supporting reproducibility and product authentication [31].

## 6 Biological properties of *P. boldus*

The biological properties of *P. boldus* have attracted increasing scientific interest due to the remarkable chemical diversity of its secondary metabolites and their potential therapeutic applications. Experimental evidence accumulated over the last decade indicates that extracts and isolated compounds from different plant organs exhibit a broad spectrum of biological activities (Table 1). However, the magnitude and nature of these activities are highly influenced by factors such as the plant tissue analyzed, extraction methodology, phytochemical composition, and experimental model employed. Therefore, a critical evaluation of the available evidence is essential to identify consistent biological trends, methodological limitations, and future research priorities.

**TABLE 1:** List of metabolites and biological activity identified in *P. boldus* extracts.

Plant tissue	Chemical class and list of metabolites	Type of extraction	Biological activity	References
Leaves	N/A	Solvent extraction, supercritical CO_2_ extraction	**Antimicrobial**: *Escherichia coli, Pseudomonas aeruginosa, Salmonella enterica, Staphylococcus aureus*, *Bacillus subtilis*, *B. cereus* **Antioxidant**: DPPH method	[49]
Leaves	N/A	Aqueous extraction	**Antimicrobial**: Production of endogenous antimicrobial peptide **Anti‐inflammatory**: secretion of TNF‐α	[50]
Leaves	N/A	Aqueous extractions combined with *Spiraea ulmaria*	**Antimicrobial**: *Staphylococcus* spp.	[51]
Leaves	N/A	Solvent extraction	**Antimicrobial**: *Staphylococcus aereus* **Antioxidant**: DPPH, ABTS, phosphomolybdenum complexation	[52]
Leaves	**Terpenoids**: α‐thujene, α‐pinene, sabinene, β‐pinene, α‐phellandrene, ocimene, limonene, linalila, α‐terpinene, 1,8‐cineole, γ‐terpinene, fenchone, terpinolene, linalool, *trans*‐sabinene hydrate, *trans*‐pinocarveol, pinocarvone, *trans*‐*p*‐menth‐1‐en‐4‐ol, α‐terpineol, ascaridole, bornyl acetate, isoascaridol **Phenolic compounds**: methyleugenol	Hydrodistillation	**Antimicrobial**: *Candida parapsilosis, C. tropicalis, C. lusitaniae, C. albicans, C. guilliermondii*	[40]
Leaves	**Terpenoids**: α‐thujene, α‐pinene, sabinene, β‐pinene, α‐phellandrene, ocimene, limonene, linalila, α‐terpinene, 1,8‐cineole, γ‐terpinene, fenchone, terpinolene, linalool, *trans*‐sabinene hydrate, *trans*‐pinocarveol, pinocarvone, *trans*‐*p*‐menth‐1‐en‐4‐ol, α‐terpineol, ascaridole, bornyl acetate, isoascaridol **Phenolic compounds**: methyleugenol	Hydrodistillation	**Antiinsecticidal**: *Aedes aegypti and Lipaphis erysimi*	[41]
Leaves	**Terpenes**: *p*‐cymene, eucalyptol, α‐terpinene **Alkaloids**: isocorydine, rogersine, boldine, reticuline, coclaurine, laurotetanine, *N*‐methylcocluarine, noisocorydine	Acid‐base liquid‐liquid partioning and spectral database	N/A	[42]
Leaves	**Phenolic compounds**: catechin, epicatechin, rutin	Ultrasound‐assisted extraction	**Antimicrobial**: *E. coli, Bacillus cereus, Candida albicans, Arthroderma crocatum* **Anti‐inflammatory**: evaluation of markers in rat prostates treated with LPS **Antioxidant**: reduction of marker of lipid peroxidation **Antiproliferative**: on human prostate carcinoma PC3 cells	[43]
Leaves	N/A	Aqueous extraction	**Antioxidant**: reduction of ROS and catalase activity **Antiobesity**: decreasing in neutral lipid, intracellular triglyceride and cholesterol accumulation **Antioxidant**: DPPH method	[53]
Leaves	**Terpenoids**: α‐pinene oxide, γ‐terpinene,β‐pinene, ρ‐cymene, (±)‐limonene, eucalyptol, trans‐sabinene hydrate, α‐terpinene, piperitone oxide, 1,3,4‐eugenol methyl ether, (−)‐β‐caryophyllene epoxide	Steam distillation and microencapsulation	**Antimicrobial**: *Penicillium spp*.	[54, 55, 56]
Leaves	**Aminoacids**: alanine, asparagine, glutamine, phenylalanine, proline, tyrosine and valine **Carboxylic acids**: acetic, citric, formic, glyceric, malic, malonic and succinic acids **Carbohydrates**: α/β−glucose, fructose and sucrose **Alkaloids**: coclaurine, *N*‐methylcoclaurine, laurolitsine, norreticuline, boldine, isoboldine, laurotenanine, reticuline *N*‐methyllaurotetanine, isocoridine and glaucine **Phenolic compounds**: luteolin‐pentosyl glycosyl rhamnose, caempferol‐3‐*O*‐glycosyl‐rhamnosyl‐rhamnose and caffeoylquinic phenolic acid glycoside	Ultrasound‐assisted extraction	N.A.	[45]
Leaves	**Alkaloids**: boldine **Phenolic compounds**: 3,4‐dihydroxybenzoic, vanillic, syringic, gallic, caffeic, ferulic, coumaric acid and rutin	Aqueous extraction	**Antioxidant**: protection against Mn toxicity in a *Drosophila melanogaster* model	[46]
Leaves	**Phenolic compounds**: rutin, kaempferol‐3‐*O*‐β, cyanidin, peonidin	Aqueous extraction	**Antioxidant**: DPPH **Antimutagenic activity**: against ethyl methane sulphonate in *Drosophila melanogaster* model	[47]
Leaves	**Alkaloids**: coclaurine, *N*‐methylcoclaurine, laurolitsine, isoboldine, boldine, reticuline, isocorydine, laurotetatine and *N*‐methyllaurotetanine, isoboldine, laurotetanine	NADES	N.A.	[12]
Leaves	**Alkaloids**: boldine	Soxhlet extraction	**Antioxidant**: DPPH	[31]
Leaves	**Alkaloids**: coclaurine, *N*‐methylcoclaurine, laurolitsine, norreticuline, boldine, isoboldine, laurotetanine, reticuline, *N*‐methyllaurotetanine, isocoridine, glaucine **Phenolic compounds**: luteolin‐pentosyl‐glycosyl‐rhamnose, caempferol‐3‐O‐glycosyl‐rhamnosyl‐rhamnose, Caffeoylquinic phenolic acid glycoside	Aqueous extraction	**Antioxidant**: DPPH, ABTS, FRAP, β‐carotene/linoleic acid	[48]
Leaves	**Phenolic compounds**: procyanidin C1 B‐type linkage, luteolin‐glucopyranosilglucopyranoside, cyanidin 3,5‐diglucoside, isorhamnetin‐*O*‐rutinoside, isorhamnetin‐*O*‐deoxyhexosylpentoside, kaempferol‐*O*‐pentosyl‐*O*‐dirhamnoside, isorhamnetin‐*O*‐dirhamnoside and luteolin‐*O*‐hexoside	Solvent extraction	**Antioxidant**: DPPH	[44]
Leaves	N.A.	Extract	**Antioxidant**: Protection against heat stress when administrated in feed	[57]
Fruits	**Alkaloids**: *N*‐methyl‐laurotetatine, boldine, higenamine isocorydine, laurolitsine **Phenolic compounds**: 3‐hydroxybenzoic acid, caffeic acid, catechin, chlorogenic acid, chrysin, quercetin, sinapic acid, syringic acid, quercetin	Solvent extraction	**Anti‐inflammatory**: in a murine macrophage model **Antioxidant** (in silico)	[10]
Fruits	**Phenolic compounds**: catechin, rutin, quercetin, epicatechin, coumaric acid **Terpenoids**: abscisic acid	Ultrasound‐assisted extraction	N.A.	[19]
Bark (single tree)	**Alkaloids**: boldine, laurolitsine, reticuline, coclaurine	Dichloromethane extraction	N.A.	[14]
Wood (single tree)	**Alkaloids**: laurotetanine, boldine, laurolitsine, reticuline, *N*‐methylcoclaurine	Dichloromethane extraction	N.A.	[14]
Root (single tree)	**Alkaloids**: laurotetanine, boldine, laurolitsine, reticuline, coclaurine	Dichloromethane extraction	N.A.	[14]
Leaves (single tree)	**Alkaloids**: laurotetanine, boldine, laurolitsine, reticuline, *N*‐methylcoclaurine, coclaurine	Dichloromethane extraction	N.A.	[14]

Abbreviation: N.A., not analyzed.

### 6.1 Antimicrobial Potential of *P. boldus*: From Phytochemical Diversity to Biological Activity

The antimicrobial activity of *P. boldus* appears to be strongly influenced by extraction technology, the physicochemical characteristics of the resulting matrix (crude extracts vs. isolated EOs), and the structural characteristics of the target microorganisms. Rather than producing a uniform inhibitory response, comparative analyses of recent studies reveal methodological patterns and unresolved discrepancies that significantly influence biological efficacy.

A major trend observed in recent years is the increasing use of AETs, particularly supercritical CO_2_ (sc‐CO_2_), to optimize the recovery of hydrophobic antimicrobial constituents. Comparative studies between conventional extraction methods and green technologies suggest that higher extraction yields or solvent polarity do not necessarily correlate with greater biological activity. Vieitez et al., demonstrated a selective susceptibility pattern in which hydrophobic fractions, including hexane extracts and sc‐CO_2_ extracts obtained at 300 bar, exhibited superior antimicrobial activity against Gram‐positive bacteria such as *Staphylococcus aureus*, *Bacillus subtilis*, and *B. cereus*, reaching a minimum inhibitory concentration (MIC) as low as 0.19 mg/mL for *B. cereus* [49].

This preferential activity toward Gram‐positive microorganisms appears consistently throughout the literature. Gram‐positive bacteria generally exhibit greater sensitivity to *P. boldus*‐derived metabolites than Gram‐negative organisms. This differential response has been associated with the structural properties of the Gram‐negative outer membrane, which limits the penetration of hydrophobic compounds such as terpenoids and long‐chain phenolics. Even under conventional extraction conditions, such as the 75% ethanolic leaf extracts evaluated by da Cruz et al., relatively elevated MIC values against *S. aureus* (6.25 mg/mL) were observed, supporting the greater efficacy frequently associated with less polar or volatile fractions [52].

However, this apparent trend becomes more complex when antimicrobial activities of aqueous extracts are considered. While antimicrobial effects are commonly attributed to lipophilic compounds, Ferrante et al., reported that aqueous leaf infusions displayed significant antibacterial activity against both Gram‐positive and Gram‐negative bacteria, with remarkably low MIC values against *Escherichia coli* (24.8 µg/mL) and *B. cereus* (78.74 µg/mL) [43].

Rather than representing a methodological inconsistency, these findings suggest the existence of alternative mechanisms mediated by hydrophilic compounds. Molecular docking analyses performed by Ferrante et al., indicated that flavonol glycosides such as rutin may interact with bacterial enzymatic targets, including nitric oxide synthase from *B. cereus*. Nevertheless, binding affinity analyses alone suggest that rutin is unlikely to fully explain the observed biological activity, supporting the possibility of synergistic interactions among multiple phenolic compounds and other bioactive metabolites [43].

Evidence for multi‐compound interactions is also observed in antifungal studies. Madrid et al., characterized EOs obtained from fresh *P. boldus* leaves collected from coastal drylands and reported strong antifungal activity against clinical isolates of *Candida* spp. Particularly notable was the activity against *C. guilliermondii*, which exhibited an MIC of 8 µg/mL, lower than that reported for conventional antifungal agents such as fluconazole and itraconazole under the evaluated conditions. The antimicrobial effect was associated with a volatile profile dominated by ascaridole (49.95%), *O*‐cymene (13.45%), and eucalyptol (9.71%), indicating that volatile fractions may operate through mechanistic pathways distinct from those of non‐volatile phenolic extracts [40].

Beyond human therapeutic applications, the antimicrobial potential of *P. boldus* is increasingly being explored in veterinary and agricultural contexts. In canine models of atopic dermatitis, Santoro et al. (2017, 2018) demonstrated that a 0.1% mixture of *P. boldus* and *Filipendula ulmaria* (Rosaceae) leaf extracts reduced *Staphylococcus* spp. colonization after 14 days of treatment. Interestingly, this reduction occurred without significant alterations in endogenous antimicrobial peptide secretion, suggesting that the observed effects may result primarily from direct antimicrobial activity rather than immune modulation [50, 51].

Similarly, agricultural studies have explored the use of *P. boldus* EOs as natural preservation agents. Girardi et al., developed microencapsulation systems through complex coacervation for controlled release of *P. boldus* EOs in stored peanut (*Arachis hypogaea*) seeds, significantly reducing fungal proliferation during storage. However, treatment also negatively affected seed germination, highlighting an important limitation in balancing antimicrobial efficacy and matrix compatibility [54, 55, 56].

Despite these promising findings, a major limitation in *P. boldus* antimicrobial research remains the gap between predictive computational approaches and experimentally validated biological outcomes. Molecular docking analyses frequently identify minor constituents as potentially strong binders due to favorable interaction energies; however, these predictions do not necessarily translate into measurable antimicrobial effects because biological activity is also influenced by concentration, compound abundance, bioavailability, and interactions within complex phytochemical matrices.

Overall, the antimicrobial literature on *P. boldus* remains limited by the extensive use of chemically uncharacterized crude extracts and insufficient standardization among studies. Future research should prioritize bioassay‐guided fractionation, standardized chemical profiling, and long‐term in vivo validation to establish reproducible structure–activity relationships and fully exploit the therapeutic and industrial potential of this endemic Chilean specie.

### 6.2 The Antioxidant‐Inflammation Nexus: Mechanistic Synergy and Multi‐Target Crosstalk in *P. boldus* Bioactivity

#### 6.2.1 Antioxidant Capacity and Neuroprotective Potential: Beyond Chemical Scavenging

The evaluation of the antioxidant properties of *P. boldus* represents one of the most extensively investigated areas within its ethnopharmacological profile. Phenolic compounds and alkaloids are considered major contributors to these biological effects [6], whereas volatile fractions from EOs, despite being primarily associated with antimicrobial activity, may also contribute to antioxidant responses to a lesser extent [16]. Nevertheless, a critical examination of the literature reveals important methodological limitations and apparent inconsistencies among published studies.

A notable discrepancy emerges when comparing the antioxidant performance of aqueous and hydroalcoholic leaf extracts across different cell‐free assays. Aqueous infusions frequently demonstrate high scavenging activity against ABTS•^+^ radicals but comparatively lower activity in DPPH• assays, a pattern that has been associated with differences in procyanidin composition [44]. Rather than representing a contradiction in the intrinsic chemistry of *P. boldus*, this variation likely reflects differences in the physicochemical characteristics and reaction mechanisms of the assays employed. The DPPH method, typically performed in organic environments, is influenced by steric accessibility and may underestimate the activity of highly hydrophilic antioxidants. In contrast, the ABTS assay can interact more efficiently with both hydrophilic and lipophilic compounds, frequently providing a broader estimation of antioxidant potential, particularly for flavonoid‐rich aqueous extracts [58].

This methodological limitation highlights an important gap in *P. boldus* antioxidant research: the extensive dependence on cell‐free chemical assays such as DPPH, ABTS, FRAP, and β‐carotene/linoleic acid systems. Although these approaches remain valuable for rapid preliminary screening of antioxidant activity, they provide only a simplified representation of biological systems and may not accurately predict physiological effects. Importantly, these assays do not adequately account for factors that critically influence in vivo activity, including gastrointestinal bioavailability, metabolic biotransformation, cellular uptake, and intracellular distribution of bioactive compounds.

To address these limitations, recent mechanistic trends have shifted toward cellular models, demonstrating that the true therapeutic value of *P. boldus* does not rely on direct, sacrificial chemical scavenging in the extracellular space. Instead, its extracts act as proactive biomodulators that bridge the gap between oxidative stress and chronic inflammation through upstream signaling pathways. At the center of this regulatory network is the modulation of the NF‐κB signaling pathway, a key regulator of pro‐inflammatory gene expression, together with activation of the Nrf2/ARE pathway, which orchestrates cytoprotective antioxidant responses [59, 60].

For instance, in differentiated 3T3‐L1 adipocytes, buffered aqueous leaf extracts reduced intracellular reactive oxygen species (ROS) level and modulated catalase activity, suggesting an influence on cellular redox homeostasis and metabolic regulation [53]. These antioxidant responses may contribute to downstream anti‐inflammatory effects observed in experimental models. In isolated rat prostates challenged with *E. coli* lipopolysaccharide (LPS), treatment with *P. boldus* extracts resulted in a significant reduction in both prostaglandin E2 (PGE2), a key mediator of inflammatory responses, and the oxidative stress biomarker 8‐iso‐PGF2α [43]. Likewise, rutin‐rich leaf extracts effectively reduced lipid peroxidation markers, including 8‐iso‐PGF2α, at the cellular level [43], supporting the hypothesis that attenuation of oxidative stress may contribute to the modulation of inflammatory pathways, including those associated with cyclooxygenase‐mediated responses.

#### 6.2.2 Deconstructing the “Silver‐Bullet” Myth: Phytochemical Synergy and Matrix Effects

Historically, ethnopharmacological studies have focused on boldine as the primary “silver‐bullet” molecule responsible for the dose‐dependent anti‐inflammatory and antinociceptive activities observed in animal models [61, 62, 63]. However, modern multi‐target pharmacology and advanced density functional theory (DFT) calculations have challenged this reductionist view, proving that the species' exceptional redox and anti‐inflammatory potential arises from a concerted electron‐donation mechanism and synergistic crosstalk within the whole complex matrix [10].

Evidence for these cooperative interactions was observed when evaluating alternative *P. boldus* organs, such as fruits. In murine macrophage models, non‐cytotoxic concentrations of *P. boldus* fruit extracts significantly attenuated inflammatory responses and were associated with modulation of NF‐κB signaling pathways [10]. Complementary computational analyses based on quantum chemical approaches further suggest that boldine and the alkaloid *N*‐methyl‐laurotetanine possess similar intrinsic antioxidant properties, which may be reinforced through interactions with structurally distinct flavonoids such as catechin, quercetin, and chrysin [10]. Collectively, these findings expand the traditional leaf‐centered perspective of *P. boldus*, highlighting fruits as an additional bioactive source whose phytochemical complexity may contribute to antioxidant and anti‐inflammatory effects through multi‐component interactions.

The crude extract demonstrated significant biological activity in vitro, a trend further supported by computational analyses where in silico molecular docking identified the minor component bornyl acetate as the most potent ligand, capable of acting against various inflammatory, or oxidative pathways, showing the lowest binding energy among all tested compounds. Nevertheless, this complex matrix behavior introduces fascinating contradictions when transitioning from in vitro to in silico environments. For instance, when testing traditional aqueous leaf extracts directly against the acetylcholinesterase (AChE) enzyme in vitro, assays have occasionally yielded negative or negligible inhibition results [48]. Conversely, molecular docking simulations reveal that isolated aporphine alkaloids, including isoboldine, boldine, coclaurine, laurotetanine, reticuline, and *N*‐methylcoclaurine, display high binding affinities toward the AChE binding pocket, with *N*‐methyl‐laurotetanine reaching an outstanding docking energy score of ‐10.1 kcal/mol [48]. This striking divergence emphasizes that while some minor components like bornyl acetate align with overall extract efficacy, other whole crude extracts may suffer from antagonistic matrix effects, steric hindrance by major fractions, or concentration thresholds in vitro. Consequently, targeted standardization or isolated fractionation remains a mandatory path forward for multi‐target neuroinflammatory and Alzheimer's research.

### 6.3 Other Biological Activities and Applied Pharmacological Evidence

EOs derived from *P. boldus* leaves exhibited significant larvicidal and insecticidal activity against *Aedes aegypti* larvae and *Lipaphis erysimi*, with median lethal doses (LD_50_) below 50 µg/mL and 60 µg/mL, respectively. Despite in silico docking studies identifying minor constituents such as bornyl acetate as high‐affinity ligands for AChE, in vitro assays did not confirm enzymatic inhibition by the whole EO. This discrepancy suggests that biological activity is primarily driven by major volatile constituents acting through alternative toxicodynamic mechanisms, while low‐abundance compounds may not reach effective functional concentrations in complex mixtures [41].

In mammalian models, oral administration of *P. boldus* extracts (100 mg/kg) promoted regenerative responses in rat liver following 70% partial hepatectomy, evidenced by a modest increase in Ki‐67 expression, a marker for cellular proliferation, although standard biochemical liver markers showed minimal variation [64]. In *Drosophila melanogaster*, aqueous extracts demonstrated antigenotoxic activity by reducing ethyl methanesulfonate‐induced mutagenicity, without exhibiting intrinsic mutagenicity, an effect attributed to phenolic compounds such as flavonoids and anthocyanins [47]. Consistently, crude aqueous extracts have shown superior protective effects compared to isolated boldine in models of manganese‐induced neurotoxicity, supporting synergistic interactions among multiple antioxidant constituents [46].

At the cellular level, aqueous extracts modulate lipid metabolism in differentiated 3T3‐L1 adipocytes, reducing intracellular triglyceride and cholesterol accumulation, although without significant effects on TNF‐α expression [53]. In human prostate carcinoma (PC3) cells, leaf extracts exhibit antiproliferative activity comparable to other medicinal plant species, including *Epilobium angustifolium* (Onagraceae) and *Phyllanthus niruri* (Phyllantaceae), suggesting a multifactorial mechanism potentially associated with phenolic compounds such as rutin, as well as additional unidentified bioactive constituents [43].

Hepatoprotective effects have also been reported in vivo. *P. boldus* leaf extracts mitigate oxidative stress, lipid peroxidation, and histopathological alterations induced by azoxystrobin fungicide exposure in rats, while modulating antioxidant gene expression. These effects were associated with compounds such as gallic acid, ellagic acid, quercetin, ferulic acid, daidzein, and rutin [65]. Similarly, in a carbon tetrachloride (CCl_4_)‐induced cirrhosis model, a multi‐herbal formulation containing *P. boldus* improved both biochemical and histopathological indicators of liver injury. However, because *P. boldus* was administered as part of a complex herbal formulation, its individual contribution to the observed hepatoprotective effects cannot be determined [66].

Finally, clinical evidence from a multicomponent herbal supplement (Fluxonorm) containing *P. boldus* extracts suggests improvement in lower urinary tract symptoms associated with benign prostatic hyperplasia, along with modulation of inflammatory markers and a favorable safety profile. However, the polyherbal nature of the formulation limits attribution of the observed effects specifically to *P. boldus*, requiring cautious interpretation of its clinical relevance as a single‐agent therapy [67].

### 6.4 Proposed Molecular Mechanisms Underlying Biological Activities

The biological activities of *P. boldus* extracts appear to result from the combined action of multiple phytochemicals acting through interconnected molecular mechanisms rather than from a single bioactive constituent. Experimental studies using the extracts indicate that their biological effects are primarily associated with modulation of oxidative stress and inflammatory responses. These extracts have been shown to reduce intracellular ROS, attenuate lipid peroxidation, and modulate the production of inflammatory mediators such as PGE_2_ [43, 53]. Collectively, these effects have been associated with modulation of redox‐sensitive signaling pathways, particularly NF‐κB, leading to reduced expression of pro‐inflammatory mediators including TNF‐α, IL‐1β, COX‐2, and iNOS [10, 53] (Figure 5A).

**FIGURE 5:**
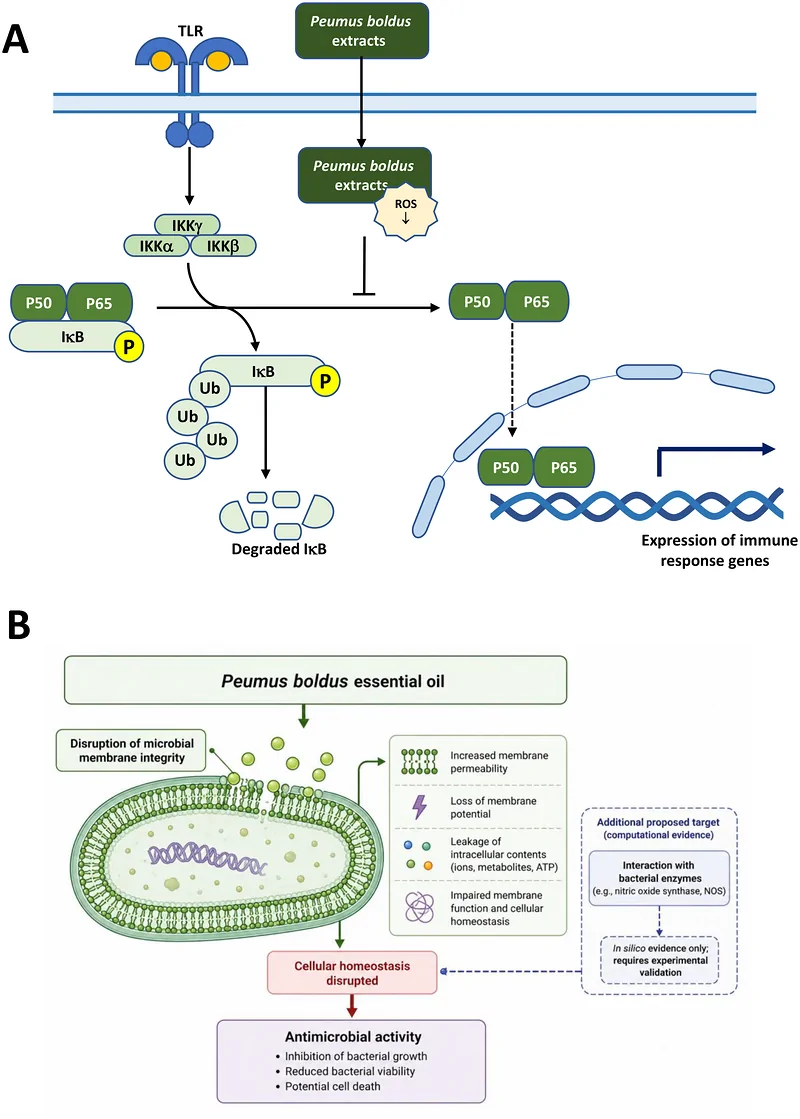
Proposed molecular mechanisms underlying the biological activities of *P. boldus*. (A) Proposed anti‐inflammatory and antioxidant mechanisms of *P. boldus* extracts. Bioactive compounds, including alkaloids, phenolic compounds, and terpenoids, may reduce ROS and inhibit NFκB signaling by limiting the nuclear translocation of the p65 subunit, leading to reduced expression of pro‐inflammatory mediators, including TNF‐α, IL‐1β, IL‐6, COX‐2, and iNOS, ultimately attenuating inflammation and oxidative damage. TLR: T cell receptor; IKK: IκB kinase complex composed by α, β and γ subunits; P50: NFκB1 subunit; P65: NFκB2 subunit; IκB: NFκB inhibitor. (B) Proposed antimicrobial mechanisms of *P. boldus* essential oil. Volatile terpenoids are thought to exert antimicrobial activity primarily through disruption of microbial membrane integrity, resulting in increased membrane permeability, loss of membrane potential, leakage of intracellular constituents, and impairment of cellular homeostasis, thereby reducing bacterial viability. Additional interactions with bacterial enzymes, such as nitric oxide synthase (NOS), have been proposed based mainly on computational studies and require further experimental validation.

The phytochemical composition of these extracts likely underlies these biological responses. Alkaloids, including boldine, laurotetanine, and *N*‐methyl‐laurotetanine, together with phenolic compounds such as rutin, catechin, quercetin, gallic acid, and ferulic acid, are considered major contributors to the antioxidant and anti‐inflammatory properties of *P. boldus* [10, 53]. However, current evidence suggests that these effects arise from additive or synergistic interactions within the phytochemical matrix rather than from the activity of individual compounds alone.

Similarly, EOs rich in volatile terpenoids, including fenchone, ascaridole, eucalyptol, and α‐terpinene, appear to exert antimicrobial activity primarily through disruption of microbial membrane integrity and interference with cellular homeostasis [49, 52]. Additional mechanisms involving interactions with bacterial enzymes, such as nitric oxide synthase, have also been proposed, although these findings are currently supported mainly by computational studies and require further experimental validation [43] (Figure 5B).

Overall, the available evidence supports a multi‐target mode of action in which complex phytochemical mixtures modulate interconnected pathways involved in oxidative stress, inflammation, apoptosis, and antimicrobial viability, thereby explaining the broad spectrum of antioxidant, anti‐inflammatory, antimicrobial, neuroprotective, hepatoprotective, nephroprotective, and cytoprotective activities attributed to *P. boldus*.

### 6.5 Translational Applications and Current Knowledge Gaps

Parallel to basic pharmacology, the translational potential of these standardized fractions is driving innovative trends across biotechnology, food science, and veterinary medicine. Solid‐liquid extractions have been optimized to isolate stable phenolic fractions (achieving 37.78 mg GAE/g) capable of encapsulating and preserving sensitive antioxidants like catechin, pyrogallol, and epigallocatechin [68]. In food science, hydroethanolic leaf extracts have been successfully integrated into gelatin films to act as active antioxidant barriers for food packaging [69]. Even in veterinary models, the administration of leaf extracts via drinking water effectively mitigated the physiological damage of heat stress in broiler chicks, improving their overall growth performance and systemic antioxidative status [57]. Despite these promising multi‐disciplinary advances, a profound operational gap persists in the literature. Historically, *P. boldus* has been used in Chilean and Mediterranean traditional medicine to alleviate conditions associated with chronic inflammation, such as rheumatic pain and urinary tract inflammation [5, 6]. Yet, most contemporary data remains confined to in silico docking profiles, in vitro enzymatic inhibitions, or short‐term veterinary/animal models. There remains an urgent need to transition these successful dual mechanisms into robust, long‐term in vivo clinical validation to fully confirm human therapeutic efficacy and establish safe therapeutic windows, ensuring that the concentrations required for dual Nrf2/NF‐*κ*B modulation do not trigger localized cytotoxic thresholds.

## 7 Biosynthesis and Aporphine Alkaloids

According to chemical structure, around of 13 classes of alkaloid exist in plants [70]. The alkaloids identified in *P. boldus* are mainly isoquinoline alkaloids, particularly aporphine alkaloids, which could originate biosynthetically from tetrahydroisoquinoline intermediates. A distinctive phytochemical characteristic of *P. boldus* is its ability to accumulate a wide range of aporphine alkaloids, including boldine, isoboldine, laurotetanine, *N*‐methyl‐laurotetanine, laurolitsine, glaucine, isocorydine, coclaurine, reticuline, and norreticuline. Other classes of alkaloids include benzylisoquinoline, proaporphine, morphinandienone, and secoaporphine alkaloids (Table 2).

**TABLE 2:** Isoquinoline alkaloids identified in *P. boldus* classified according to their chemical structure.

Chemical sub clase	Alkaloid name
Aporphine alkaloids	Isoboldine, laurolitsine, boldine, laurotetanine, *N*‐methyl‐laurotetanine, norglaucine, glaucine, isocorydine, norisocorydine, isocorydine *N*‐oxide, 6α,7‐dehydroboldine
Benzylisoquinoline alkaloids	Higenamine, coclaurine, *N*‐methylcoclaurine, reticuline, nor‐reticuline
Proaporphine alkaloids	Glaziovine, pronuciferine
Morphinandienone alkaloids	Pallidine, sinoacutine
Secoaporphine alkaloids	Secoboldine, N‐methylsecoboldine

The biosynthesis of isoquinoline alkaloids in *P. boldus* begins with L‐tyrosine through the benzylisoquinoline alkaloid pathway. L‐tyrosine is converted into dopamine and 4‐hydroxyphenylacetaldehyde, which subsequently condense via norcoclaurine synthase (NCS) to form (S)‐norcoclaurine, the universal precursor of benzylisoquinoline alkaloids. Subsequent methylation, hydroxylation, and oxidation reactions catalyzed by methyltransferases, cytochrome P450 monooxygenases, and oxidoreductases convert this intermediate into (S)‐reticuline, the central branch‐point intermediate in isoquinoline alkaloid biosynthesis. While benzylisoquinoline alkaloids such as coclaurine, *N*‐methylcoclaurine, higenamine, and reticuline itself constitute the early products of this pathway, (S)‐reticuline undergoes oxidative phenolic coupling and further structural modifications to generate the major structural subclasses identified in *P. boldus*, including aporphine, proaporphine, morphinandienone, and secoaporphine alkaloids, with boldine representing the predominant aporphine alkaloid [71]. The occurrence of multiple alkaloid classes derived from (S)‐reticuline may provide ecological and pharmacological advantages. From a biological perspective, increased alkaloid diversity enhances plant defense against herbivores, pathogens, and other environmental stresses by expanding the range of bioactive metabolites. From a pharmacological point of view, structural diversity may broaden the spectrum of biological activities and increase the likelihood of synergistic interactions, highlighting *P. boldus* as a valuable source of bioactive compounds.

### 7.1 Structure–Activity Relationships in Alkaloids

The biological activity of *P. boldus* is closely tied to the structural features of its major alkaloids. In boldine and related aporphine alkaloids, the presence of phenolic hydroxyl groups is particularly relevant because these groups can donate electrons or hydrogen atoms, thereby stabilizing ROS and contributing to antioxidant activity [10, 70]. This structural feature may explain, at least in part, the protective effects reported in experimental models of oxidative stress.

Methoxy substituents, which are also common in aporphine alkaloids, may modulate activity by modifying lipophilicity, membrane permeability, and interactions with molecular targets [70]. Small differences in the number and position of hydroxyl and methoxy groups among boldine, isoboldine, laurotetanine, and *N*‐methyl‐laurotetanine are likely to affect antioxidant capacity, bioavailability, and affinity toward proteins involved in oxidative stress and inflammatory pathways. As a result, even structurally similar aporphines may differ in their pharmacological performance.

Comparative studies on aporphine and benzylisoquinoline alkaloids provide direct structural evidence for the biological activities reported in *P. boldus* extracts. Using a Fe^2+–^cysteine‐induced microsomal lipid peroxidation assay, Cassels et al., compared a panel of aporphine derivatives and found boldine, bearing a free hydroxyl at C1, to be nearly twice as active as its regioisomer isoboldine, indicating that the position of the phenolic hydroxyl on the aporphine skeleton, rather than its mere presence, governs the extent of radical‐scavenging activity [72]. A parallel trend was observed among benzylisoquinoline analogues, where *O*‐methylation of the phenolic hydroxyls markedly reduced activity, whereas laudanosoline alkaloid, which retains an unhindered catechol, reaches the highest potency of the series. Quaternisation of the aporphine nitrogen, as in *N*‐methylboldinium, similarly lowered radical‐scavenging capacity relative to boldine, consistent with a mechanism in which the tertiary amine lone pair stabilizes the phenoxy radical formed upon hydrogen donation [72, 73]. Taken together, these findings suggest that the antioxidant potential attributed to the alkaloid fraction of *P. boldus* depends less on the aporphine core *per se* than on the number, position, and methylation state of its phenolic substituents, together with the availability of the nitrogen lone pair. These structural requirements that merit consideration when boldine‐enriched extracts or synthetic analogues are proposed for pharmaceutical development.

From a pharmaceutical perspective, these structure–activity relationships highlight the importance of comprehensive phytochemical characterization and the identification of reliable bioactive chemical markers for standardization and quality control. Several knowledge gaps still limit the translational potential of *P. boldus*. Few studies have integrated quantitative metabolomic profiling (e.g., HRMS‐ESI and UHPLC‐MS/MS) with bioactivity‐guided fractionation to identify the metabolites responsible for the reported pharmacological effects. In addition, the structure–activity relationships of aporphine alkaloids remain poorly understood, particularly regarding the influence of hydroxylation, methoxylation, and stereochemistry on biological activity. The impact of geographic origin, environmental conditions, and fruit developmental stage on phytochemical composition and therapeutic efficacy has not been systematically evaluated under standardized conditions. Furthermore, most available evidence is based on in vitro assays and rodent models, whereas well‐designed clinical studies investigating pharmacokinetics, dose–response relationships, efficacy, and safety in humans remain scarce [74].

### 7.2 Tissue‐Specific Phytochemical Variability and Ecological Modulation

Research conducted by Fuentes‐Barros et al., revealed a highly complex and tissue‐specific alkaloid distribution in *P. boldus*, identifying eighteen distinct alkaloids, including four compounds (secoboldine, *N*‐methylsecoboldine, glaucine, and norreticuline) previously unreported in the specie. Their findings demonstrate clear organ‐dependent specialization in secondary metabolism: while boldine was the dominant alkaloid in bark tissue, leaves were characterized by a distinct profile in which *N*‐methyl‐laurotetanine, laurotetanine, and coclaurine predominate. In contrast, laurolitsine was identified as the major alkaloid in wood and roots. Based on the analysis of 130 individual trees, the study further shows that leaf alkaloid composition is highly variable and significantly influenced by both tree age and sex, evidencing strong intraspecific chemical plasticity. Collectively, these results challenge the traditional assumption that leaves are the principal pharmacologically representative tissue and suggest that widely used foliar preparations may not accurately reflect the high boldine concentrations historically attributed to the species’ therapeutic value [14].

In a complementary study, Fuentes‐Barros et al., expanded this framework to a broader ecological and phytochemical analysis of Chilean populations, employing UHPLC‐MS/MS for non‐volatile metabolites and GC‐MS for EO characterization. Their results indicate that wild populations from north‐central Chile contain the highest concentrations of alkaloids (including boldine, isocorydine, and higenamine) and phenolic compounds, with a general decline observed in more southern populations. Experimental cultivation further demonstrated a clear environmentally driven metabolic trade‐off: increased light exposure enhanced polyphenol accumulation, whereas shaded conditions favored alkaloid biosynthesis. In EOs profiles, wild individuals were characterized by higher levels of ascaridole, while cultivated plants accumulated its precursor α‐terpinene as a dominant constituent. Overall, the study concludes that phytochemical variability in *P. boldus* is governed by a complex interaction of environmental factors—including light availability, rainfall, and plant developmental stage—rather than latitude alone, highlighting the ecological plasticity of its secondary metabolism [13].

Beyond leaves and bark, fruits also represent a chemically distinct and biologically relevant tissue. Otero et al., showed that fruit‐derived extracts obtained through methanolic extraction of macerated material, followed by freeze‐drying, centrifugation, and filtration, exhibit a diverse phytochemical profile. UHPLC‐MS/MS analysis identified alkaloids such as *N*‐methyl‐laurotetanine, boldine, higenamine, isocoridine, and laurolitsine, alongside phenolic compounds including syringic acid, chlorogenic acid, sinapic acid, caffeic acid, catechin, chrysin, quercetin, and 3‐hydroxybenzoic acid [10]. Complementary HPLC‐DAD analyses further confirmed the presence of catechin, rutin, quercetin, epicatechin, coumaric acid, and abscisic acid, with epicatechin and catechin identified as the most abundant phenolic constituents [19]. Collectively, these findings position fruits as an additional and underexplored phytochemical reservoir within the species, exhibiting a distinct alkaloid–phenolic signature compared to vegetative tissues.

## 8 Toxicological Assessment of *P. boldus*: From Experimental Evidence to Clinical Safety

Despite the widespread traditional use of *P. boldus* in herbal medicine, the assessment of its toxicological safety has received increasing attention in recent years, whose results were summarized in Table 3.

**TABLE 3:** Toxicological and protective effects associated with *P. boldus*.

Type of evidence	Type of extract	Key findings	References
Systematic review of preclinic studies	Diverse types of extracts	Liver damage and other systemic effects, including teratogenicity and neurological changes, have been reported, in addition to liver, kidney, and hematological disorders at high doses or prolonged exposures.	[75]
Systematic review of human clinical cases	Diverse types of extracts	Very low risk, with only three reported cases of adverse effects or interactions.	[76]
In vitro cytotoxicity study	Essential oils and commercial infusions	Essential oils exhibit high cytotoxicity, greater than infusions.	[11]
Clinical case study	Infusion of leaves	87‐year‐old male with hepatotoxicity: marked elevation of liver enzymes + jaundice. Complete improvement after discontinuing boldo.	[77]
Review of clinical cases	Herbal preparations and others	It reports cases of liver damage induced by herbal remedies, including liver injury associated with boldo laxatives.	[78]
Clinical cases	Herbal mixtures	Plants used in weight control mixtures, including formulations that may incorporate *P. boldus*, are associated with liver damage in consumers.	[79]
Chemical analysis and risk assessment	Herbal tea	In at least one brand: arsenic exceeds limits (Codex/MERCOSUR). Non‐carcinogenic risk indices <1, but further evaluation is recommended.	[80]
Phytochemical and regulatory review	Essential oil	Ascaridole content highly variable across chemotypes (∼9% to >50% of essential oil); EU monograph requires quantification of ascaridole in herbal preparations.	[81, 82]
Regulatory monograph (posology)	Herbal tea/dry extract (5:1)	Recommended doses: 1–2 g tea, 2–3×/day, or 200–400 mg dry extract, 2×/day (max. 400–800 mg/day); duration ≤2 weeks without medical advice; no overdose cases formally reported.	[81]
Clinical case reports	Oral preparations (tablets/infusion)	Increased INR with warfarin (boldo plus fenugreek); decreased tacrolimus levels in a renal transplant patient, suggesting CYP3A4/3A5 induction.	[83, 84]
Regulatory monograph / preclinical reproductive toxicity	Dry ethanolic extract/boldine	Use not recommended during pregnancy/lactation; fetal anatomical alterations and abortion observed in rats at high doses.	[81]

A recent systematic review of herbal medicines derived from *P. boldus* administered systemically reported that, in addition to significant changes in hepatocytes and liver weight, effects such as alterations in hepatic fat deposition, teratogenic potential, reduced motor activity, and decreased dopaminergic neurons were also observed in animal models, suggesting that systemic use of *P. boldus* preparations may present significant toxicological risks that require further evaluation. The authors also highlight the presence of liver, kidney, and hematological disorders at high concentrations or after prolonged treatment, underscoring the need to consider toxicological parameters in its pharmacological and nutraceutical use [75]. According to the systematic review done by Di Lorenzo et al., *P. boldus* was one of the 66 botanical samples analyzed for safety. The study identified a total of three scientific papers documenting events related to its consumption: one report described a direct adverse effect, while two reported interactions with conventional drugs or nutrients. Unlike more frequently documented plants such as *Ginkgo biloba* (Ginkgoaceae) or *Glycine max* (Fabaceae), boldo was not included in the detailed “top 14” analysis because it had fewer than ten causality‐assessed reports in the literature [76].

In a comparative analysis of EOs and commercial teas from *P. boldus*, the toxicity of these preparations was estimated using LD_50_ values. The results showed that the EOs presented high cytotoxicity (LD_50_< 200 ppm) in the tests used, indicating that boldo EOs can be highly cytotoxic depending on their form of preparation and concentration [11]. From a toxicological perspective, these results reinforce the need to clearly differentiate between extract types and consumption matrices, as the higher concentration of lipophilic bioactive metabolites in EOs could explain their greater cytotoxic potential compared to traditionally used aqueous infusions. Likewise, these findings underscore the importance of establishing safe limits of use and conducting specific toxicological evaluations for each formulation prior to its therapeutic or nutraceutical application.

In a documented clinical case, an 87‐year‐old man who regularly consumed *P. boldus* infusions presented liver toxicity, including a significant elevation of liver enzymes and the development of jaundice. After discontinuing the infusion intake, complete resolution of the signs and symptoms were observed, suggesting a temporal and probable relationship between the use of boldo and hepatotoxicity in older adults [77]. Although this is an isolated report, this type of clinical evidence is particularly relevant as it highlights that even traditional preparations considered safe, such as herbal teas, could induce adverse liver effects in vulnerable populations or under specific conditions of prolonged use or high dosage. Likewise, reviews on liver damage induced by herbal remedies have reported cases of hepatotoxicity associated with the use of *P. boldus* preparations, including a case of liver injury related to herbal laxatives containing boldo, which highlights the possibility of liver damage associated with prolonged or indiscriminate use of herbal products containing this species [78]. In toxicity studies associated with commercial herbal medicinal product formulations, it has been documented those plants used in weight control mixtures, including those whose composition may incorporate *P. boldus*, are linked to liver damage in consumers. This highlights the need to carefully evaluate the hepatotoxicity of herbal preparations and to consider the risks that may accompany the use of natural products without strict regulatory control [79]. This concern is reinforced by the considerable variability in ascaridole and alkaloid content reported across commercial *P. boldus* preparations, underscoring the broader need for standardized dosing and quality control discussed below [14, 81].

In addition to the intrinsic effects of its metabolites, the safety of *P. boldus* infusions may also be compromised by environmental contaminants. A study evaluating the concentrations of arsenic, cadmium, and lead in herbal infusion bags marketed in Tacna, Peru, including products containing *P. boldus*, reported that arsenic levels in boldo infusions exceeded the limits established by Codex Alimentarius and MERCOSUR in at least one commercial brand. Although the calculated non‐carcinogenic risk indices were less than 1, suggesting a low risk under the conditions evaluated experimentally, the authors emphasized the need for additional studies to more accurately assess the potential health risks associated with the consumption of these herbal infusions. These findings highlight that the toxicological profile of *P. boldus* preparations depends not only on their phytochemical composition, but also on product quality and environmental contamination, reinforcing the importance of quality control and strict regulatory oversight of herbal products [80].

Among the constituents of *P. boldus* EO, ascaridole, a monoterpene endoperoxide, deserves specific attention as a potentially toxic compound whose proportion varies considerably across reported chemotypes, ranging from approximately 9% under standard hydrodistillation conditions to over 50% of the total oil in other phytochemical surveys [82]. Owing to this variability and its associated toxicity, the European Union herbal monograph on *P. boldus* folium specifically requires that ascaridole levels in herbal preparations and herbal medicinal products be determined and monitored as part of pharmaceutical quality control [81].

Regarding dosage and therapeutic margin, the European Union herbal monograph establishes posology guidelines for traditional use: 1–2 g of comminuted herbal substance as an infusion taken two to three times daily, or 200–400 mg of a 5:1 aqueous dry extract per dose, up to a daily dose of 400–800 mg, restricted to adults and with treatment duration limited to two weeks without medical consultation [81]. No cases of overdose have been formally reported, but the absence of dedicated pharmacokinetic and dose‐escalation studies prevents a precise definition of the therapeutic margin, representing a relevant gap for the safe pharmacological use of this species [81].

Although the European Union herbal monograph currently reports no formally established interactions with other medicinal products [81], isolated clinical case reports suggest that *P. boldus* extracts may interact with drugs of narrow therapeutic index. An increase in the international normalized ratio (INR) was documented in a patient on warfarin therapy who concomitantly consumed *P. boldus* and *Trigonella foenum‐graecum*, an effect attributed to the coumarin content of both botanicals [83]. Separately, a renal transplant recipient receiving tacrolimus presented subtherapeutic immunosuppressant levels while taking *P. boldus*, which normalized after its discontinuation, suggesting a possible interaction mediated by induction of cytochrome P450 3A4/3A5 isoenzymes [84]. These reports, although limited to single cases, highlight the need for caution and clinical monitoring when *P. boldus* preparations are combined with anticoagulants or immunosuppressants.

Safety during pregnancy and lactation has not been established for *P. boldus*, and its use is not recommended during these periods according to current regulatory guidance [81]. This recommendation is supported by preclinical reproductive toxicity studies, in which oral administration of a dry ethanolic extract of *P. boldus* leaf and of boldine to pregnant rats produced fetal anatomical alterations and isolated cases of abortion at high doses [81]. No fertility data are currently available for this species.

It should be noted that most toxicological studies have been conducted using leaf extracts or EOs, whereas information regarding the safety profile of fruit‐derived preparations remains scarce. Therefore, toxicological findings obtained from other plant organs should not be directly extrapolated to fruit extracts without further experimental validation.

## 9 Discussion

The relationship between extraction conditions, chemical composition, and antimicrobial activity is well illustrated by the work of Vieitez et al., who demonstrated that higher extraction efficiency does not necessarily translate into stronger biological activity. In their study, increasing solvent polarity enhanced metabolite recovery; however, antimicrobial assays revealed that hydrophobic extracts (e.g., hexane maceration and sc‐CO_2_ fractions) exhibited greater inhibitory activity than polar extracts such as 75% aqueous ethanol. Importantly, the susceptibility pattern also differed between bacterial groups, with Gram‐positive strains showing higher sensitivity than Gram‐negative bacteria, which is consistent with the structural barrier imposed by the outer membrane in Gram‐negative organisms that limits permeability to lipophilic compounds [49]. Complementarily, Ferrante et al., provided evidence that antimicrobial effects may parallel, at least partially, the qualitative composition of the extract and its total phenolic content. Their chemical analyses revealed complex phenolic profiles containing both identified and yet‐unidentified metabolites, suggesting that antimicrobial activity may result from the combined action of multiple compounds rather than from a single predominant molecule. Notably, molecular docking indicated that direct ligand‐target interactions would not fully explain the observed effects, reinforcing the plausibility of alternative or multi‐target mechanisms [43]. From a safety and translational perspective, the antimicrobial activity reported by this author was observed at concentrations below the LC_50_ threshold determined using the *Artemia salina* lethality assay, supporting preliminary biocompatibility of the extracts under the evaluated conditions. Taken together, these studies highlight that the antimicrobial potential of *P. boldu*s cannot be interpreted without considering extraction‐driven chemical variability, and they emphasize the need for standardized extraction protocols, chemical fingerprinting, and bioassay‐guided fractionation to identify reproducible antimicrobial signatures and metabolite interactions responsible for activity

EOs represent another major bioactive fraction of *P. boldus*, typically enriched in lipophilic terpenoids and other molecules [16]. Among these, fenchone has been associated with anti‐inflammatory, antioxidant, and antinociceptive properties [85, 86]. It has also shown antimicrobial effects against *Candida* spp. at concentrations of 32 and 64 µg/µL [87]. In a gastrointestinal context, Araruna et al., reported that fenchone promoted ulcer protection and healing through combined oxidative stress reduction (restoration of GSH and SOD altogether reduction of MDA and MPO) and immune modulation (decreased IL‐1β and TNF‐α, increased IL‐10) [86]. Additionally, in a chronic joint inflammation model, fenchone reduced mRNA expression of inflammatory mediators including inducible nitric oxide synthase (*iNOS)*, *IL‐17*, *IL‐1β*, *TNF‐α* and cyclooxygenase‐2 (*COX‐2*) mRNAs, suggesting a mechanistic basis involving iNOS and COX‐2 related pathways [85]. Importantly, safety considerations must accompany these bioactivity claims, due to Bashir et al., reported that acute oral exposure to 2000 mg/kg fenchone caused no mortality in rats (LD_50_> 2000 mg/kg), although biochemical changes and oxidative stress alterations were detected, highlighting the need for subchronic and chronic toxicological evaluations [88]. Furthermore, EO activity should not be interpreted as driven by a single dominant terpenoid, as different chemotypes enriched in alternative monoterpenes such as ascaridole have also demonstrated antimicrobial activity. Thus, metabolites may contribute significantly but likely operate within a broader network of synergistic interactions, emphasizing the relevance of systematic synergy testing and fractionation strategies.

Beyond complex mixtures, boldine remains an important pharmacological reference compound, particularly due to its multi‐target effects across different experimental systems. Boldine is a promising natural resource for the treatment of gastric ulcers, as it strengthens the mucosal defense mechanisms and combats oxidative stress without altering gastric acidity through the proton pump [89]. It has been shown to protect against estrogen deficiency‐induced bone loss by inhibiting osteoclastogenesis via impairment of the AKT signaling pathway [90], and has also been proposed as a neuroprotective candidate in Alzheimer's related models, where it interfered with β‐amyloid aggregation and stabilized mitochondrial function while mitigating ROS and Ca^2^^+^ dysregulation in HT22 cells [91]. In addition, Silva et al., reported that boldine modulated hepatic glucose metabolism in perfused rat liver by inhibiting gluconeogenesis and stimulating glycolysis while reducing mitochondrial ROS generation without compromising mitochondrial respiration, suggesting potential relevance for metabolic regulation [92]. Boldine has demonstrated nephroprotective effects against cisplatin‐induced toxicity in renal models. This is related to the modulation of oxidative stress, the regulation of caspase‐3, and the decrease in proinflammatory markers such as iNOS and TNF‐α, contributing to the preservation of renal structure and function [93].

Besides, this molecule has been proposed to ameliorate the oxidative damage of kidney in renovascular hypertension (RVH), activated by the renin‐angiotensin system (RAS). RAS is a key player by activating transforming growth factor (TGF‐β) leading to renal fibrosis. Results of Gómez & Velarde, 2018 declares that this molecule could had a beneficial effect in a 2K1C hypertensive rat model. In addition, increase levels of angiotensin II could lead to inflammation and ROS increase. Contributing also to renal fibrosis. Boldine could prevent the renal damage by affecting the RAS system [94]. Other study involved the potential of photobiomodulation (near‐infrared light stimulation) and boldine as non‐invasive treatments for diabetic retinopathy, a condition characterized by oxidative stress, inflammation, and mitochondrial dysfunction in the retina. Utilizing both obese mouse models and retinal explants, the researchers identified that high glucose levels primarily damage the mitochondria within photoreceptor cells, showing morphological changes. The results demonstrated that boldine effectively reduced oxidative‐nitrosative stress, while photobiomodulation improved mitochondrial metabolism, though the light therapy alone was insufficient to prevent structural damage [95].

These results reinforce the hypothesis that boldine acts as a pleiotropic molecule, capable of modulating different interconnected biological systems, all of which are related to the regulation of oxidative stress and inflammatory responses. Moreover, its potential adjuvant and biotechnological applications have been proposed, both to mitigate the toxicity associated with certain pharmacological treatments and for the development of nutraceutical and dermocosmetic formulations. Overall, these findings support the notion that boldine contributes to *P. boldus* bioactivity through convergent antioxidant and anti‐inflammatory mechanisms; however, given its relatively low abundance in leaves compared to other alkaloids, its role in herbal preparations should be interpreted as part of a broader phytochemical synergy rather than as a single explanatory factor.

Finally, in murine models, the administration of boldine encapsulated in PLGA nanoparticles showed a significant reduction in cisplatin‐induced cellular and tissue toxicity, reflected in lower cell death, decreased DNA damage, and modulation of oxidative stress markers in normal cells [96]. In albino Wistar rats with hepatocellular carcinoma induced by diethylnitrosamine, boldine administration protected the liver against the accumulation of ROS by enhancing the activity of antioxidant enzymes such as SOD and CAT, modulated detoxification capacity through phase I and II enzymes, and reduced proliferative markers such as PCNA and Ki‐67. It also modulated key cell cycle proteins (p21, p27, cyclin D1, CDK4, cyclin E1, CDK2) [97] and the expression of genes and proteins involved in mitochondrial apoptosis (Bcl‐2, Bax, cleaved caspase‐3), correlated with histopathological improvements in liver tissue. These results suggest a possible chemopreventive and therapeutic role in the progression of experimental liver lesions [98, 99].

Recent evidence indicates that boldine is neither the predominant alkaloid in leaves, wood, and fruits nor necessarily the main driver of biological effects in herbal preparations [10, 13, 14]. In fact, other isoquinoline alkaloids, such as laurotetanine, *N*‐methyl‐laurotetanine, isocoridine, and norisocoridine, have been reported as major constituents in this species, supporting the view that *P. boldus* bioactivity cannot be attributed to a single metabolite [10, 13]. This compositional evidence is consistent with the hypothesis that medicinal effects may result from additive or synergistic interactions among multiple compounds present at higher proportions than boldine itself [10, 33]. Moreover, phenolic constituents such as gallic acid, quercetin, ferulic acid, and rutin may also contribute substantially to the antioxidant and anti‐inflammatory profile of *P. boldus* extracts, reinforcing a multi‐compound, multi‐target mode of action.

An important limitation of the currently available evidence is the lack of clinical studies evaluating the therapeutic effects of *P. boldus* in humans. Much of the evidence comes from in vitro studies and animal models, which provide valuable information about the mechanisms of action but do not necessarily predict clinical effectiveness in humans. A limited number of clinical trials have evaluated multi‐component herbal formulations that included *P. boldus* along with other medicinal species. For example, favorable results were observed in patients with lower urinary tract symptoms associated with benign prostatic hyperplasia treated with an herbal combination [67]. More recently, a clinical trial conducted in pediatric patients undergoing urinary lithotripsy showed a significant reduction in residual microlithiasis after administering an herbal formulation composed of *P. boldus, Herniaria hirsute* (Caryophyllaceae)  and *Arctostaphylos uva‐ursi* (Ericaceae), with no adverse effects reported during follow‐up [100]. However, because *P. boldus* was administered in combination with other plant species, it is difficult to determine its specific contribution to the observed effects. As a result, well‐designed, randomized clinical trials that evaluate standardized *P. boldus* preparations are needed to confirm its effectiveness and safety, as well as to establish optimal doses and assess its long‐term effects in humans.

Importantly, the pharmacological potential of *P. boldus* must be interpreted alongside its toxicological evidence. Although several experimental studies report protective effects of boldo‐derived compounds under controlled conditions, evidence from toxicological evaluations and clinical observations indicates that herbal formulations containing *P. boldus* may be associated with hepatic injury, particularly when consumed indiscriminately, at high doses, or for prolonged periods. In this context, the documentation of liver damage associated with commercial herbal medicinal products, including weight‐control mixtures that may incorporate *P. boldus*, reinforces the need for rigorous safety assessment and regulatory surveillance of phytotherapeutic preparations [79]. Therefore, defining safe exposure windows, standardized dosing regimens, and quality control criteria across different matrices (infusions, extracts, and EO) remains a critical gap for the safe translational use of *P. boldus*.

## 10 Conclusion

The integrative analysis of *P. boldus* confirms its status as a high‐value medicinal resource whose therapeutic versatility is dictated by its unique chemical composition. The presence of the isoquinoline alkaloid boldine, alongside a complex volatile fraction containing ascaridole, 1,8‐cineole, and phenolic compounds, serves as the foundation for its multi‐target biological activity. From a physiological perspective, the plant demonstrates a powerful antioxidant activity by neutralizing free radicals and preventing oxidation status in the cell. This trait is complemented by a significant anti‐inflammatory action that modulates cellular pathways to reduce tissue damage. Furthermore, its antimicrobial activity, show efficacy against various bacterial and fungal strains, and documents other activities such as its traditional role as a protective in several models. However, the toxicology and safety profile of boldo necessitates a rigorous and cautious approach to its clinical application. While the incidence of adverse effects in humans remains statistically low under traditional use, the evidence of hepatotoxicity is substantial when the plant is consumed in high doses, in concentrated EO forms, or over prolonged periods. In conclusion, *P. boldus* is a highly effective pharmaceutical agent for treating oxidative stress, inflammation, and antimicrobial. Nevertheless, to ensure a favorable benefit‐risk ratio, its use must be standardized and regulated. Future pharmaceutical development should prioritize the isolation of bioactive compounds to reduce the potential risks associated with complex extracts. In addition, public health recommendations should discourage self‐medication with concentrated *P. boldus* preparations and recommend caution in patients with pre‐existing liver disease and in pregnant women.

## Author Contributions

**Marta Rodríguez‐Ceballos, Valentina Jara, Sofía Díaz‐Cornejo, Valentina Morales, Rómulo Santelices‐Moya, Miguel Aguilera, Cristian Atala, María Carolina Otero, and Felipe Gordillo‐Fuenzalida**: writing – review and editing. All authors have read and agreed to the published version of the manuscript.

## Funding

The authors have nothing to report.

## Conflicts of Interest

The authors declare no conflicts of interest.

## Use of Generative AI and AI‐assisted Technologies

Generative AI was used to create Figures 1 and 5B and to assist with substantial editing and restructuring of the manuscript. The authors reviewed and edited the AI‐assisted content and take full responsibility for the final content of the manuscript and figures.

## Data Availability

Data sharing not applicable to this article as no datasets were generated or analysed during the current study.

## References

[1] R. Rodríguez , C. Marticorena , D. Alarcón , et al., “Catálogo de las Plantas Vasculares de Chile,” Gayana Botánica 75 (2018): 1–430, 10.4067/S0717-66432018000100001.

[2] F. Luebert and P. Pliscoff , Sinopsis Bioclimática y Vegetacional de Chile, 2nd ed. (Editorial Universitaria, 2018), 384.

[3] Instituto Forestal (Chile). Boldo (*Peumus boldus* Mol.) Avances en la Investigación para el Desarrollo de Modelos Productivos Sustentables (INFOR; FIA, 2018).

[4] C. D. Machado , V. L. P. dos Santos , and R. S. Novak , “Contributions of Trichome Micromorphology to the Characterization of Species Traded as “Boldo”,” Flora 279 (2021): 151827, 10.1016/j.flora.2021.151827.

[5] S. Cordero , F. Gálvez , and L. Abello , Usos Tradicionales de la Flora de Chile Vol. 1 (Ediciones Botánicas, Editorial Planeta de Papel Ltda, 2020), 408.

[6] B. Salehi , J. Sharifi‐Rad , J. Herrera‐Bravo , et al., “Ethnopharmacology, Phytochemistry and Biological Activities of Native Chilean Plants,” Current Pharmaceutical Design 27 (2020): 953–970, 10.2174/1381612826666201124105623. 33234091

[7] M. C. Otero , J. A. Fuentes , C. Atala , et al., “Antimicrobial Properties of Chilean Native Plants: Future Aspects in Their Application in the Food Industry,” Foods 11 (2022): 1763, 10.3390/foods11121763. PMC9222376 35741959

[8] M. Pino and T. D. Dillehay , “Monte Verde II: An Assessment of New Radiocarbon Dates and Their Sedimentological Context,” Antiquity 97 (2023): 524–540, 10.15184/aqy.2023.32.

[9] INFOR . “Estadísticas Forestales [Forest Statistics],”, Accessed August 9, 2025, https://wef.infor.cl/.

[10] C. Otero , S. Miranda‐Rojas , F. M. Llancalahuén , et al., “Biochemical Characterization of *Peumus Boldus* Fruits: Insights of its Antioxidant Properties Through a Theoretical Approach,” Food Chemistry 370 (2022): 131012, 10.1016/j.foodchem.2021.131012. 34500293

[11] W. F. M. de Souza , X. M. Mariano , and J. L. Isnard , “Evaluation of the Volatile Composition, Toxicological and Antioxidant Potentials of the Essential Oils and Teas of Commercial Chilean Boldo Samples,” Food Research International 124 (2019): 27–33. 10.1016/j.foodres.2018.12.059 31466647

[12] J. Torres‐Vega , S. Gómez‐Alonso , J. Pérez‐Navarro , and E. Pastene‐Navarrete , “Green Extraction of Alkaloids and Polyphenols from *Peumus boldus* Leaves with Natural Deep Eutectic Solvents and Profiling by HPLC‐PDA‐IT‐MS/MS and HPLC‐QTOF‐MS/MS,” Plants 9 (2020): 242, 10.3390/plants9020242. PMC7076633 32069868

[13] G. Fuentes‐Barros , J. Echeverría , C. Mattar , et al., “Phytochemical Variation of Wild and Farmed Populations of Boldo (*Peumus boldus* Molina),” Journal of Applied Research on Medicinal and Aromatic Plants 35 (2023): 100502, 10.1016/j.jarmap.2023.100502.

[14] G. Fuentes‐Barros , S. Castro‐Saavedra , L. Liberona , et al., “Variation of the Alkaloid Content of *Peumus boldus* (boldo),” Fitoterapia 127 (2018): 179–185, 10.1016/j.fitote.2018.02.020. 29454020

[15] S. Cordero , L. Abello , and F. Gálvez , Plantas Silvestres Comestibles y Medicinales de Chile y Otras Partes del Mundo: Guía de Campo (Ediciones Corporación Chilena de la Madera, 2017).

[16] X. M. Mariano , W. F. M. de Souza , C. B. Rocha , and R. F. A. Moreira , “Bioactive Volatile Fraction of Chilean Boldo (*Peumus boldus* Molina)—An Overview,” Journal of Essential Oil Research 31 (2019): 474–486, 10.1080/10412905.2019.1617797.

[17] F. Santibáñez , Atlas Agroclimático de Chile. Estado Actual y Tendencias del Clima. Tomo III: Regiones de Valparaíso, Metropolitana, O'Higgins y Maule (Santiago, 2017).

[18] IDE‐MINAGRI . Infraestructura de Datos Espaciales del Ministerio de Agricultura [Spatial Data Infrastructure of the Ministry of Agriculture]. Accessed November 2025. https://ide.minagri.gob.cl.

[19] P. Velásquez , M. I. Sandoval , A. Giordano , M. Gómez , and G. Montenegro , “Nutritional Composition and Polyphenolic Content of Edible *Peumus boldus* Mol. Fruits,” Ciencia E Investigación Agraria 44 (2017): 64–74.

[20] I. A. V. Miranda , P. Becerra , et al., “Traits of Perch Trees Promote Seed Dispersal of Endemic Fleshy‐Fruit Species in Degraded Areas of Endangered Mediterranean Ecosystems,” Journal of Arid Environments 170 (2019): 103995, 10.1016/j.jaridenv.2019.103995.

[21] V. Bobrov , A. A. Iurmanov , and D. H. Lorence , “Phylogenetic Biogeography of the Family Monimiaceae,” Geography, Environment, Sustaintability 10 (2017): 16–26, 10.24057/2071-9388-2017-10-4-16-26.

[22] K. Peña‐Rojas , S. Donoso , R. Gangas , S. Durán , and D. Ilabaca , “Efectos de la Sequía en las Relaciones Hídricas, Crecimiento y Distribución de Biomasa en Plantas de *Peumus boldus* Molina (Monimiaceae) Cultivadas en Vivero,” Interciencia 43 (2018): 36–42.

[23] M. A. Yáñez , S. E. Espinoza , C. R. Magni , and E. Martínez‐Herrera , “Early Growth and Physiological Acclimation to Shade and Water Restriction of Seven Sclerophyllous Species of the Mediterranean Forests of Central Chile,” Plants 13 (2024): 2410. 10.3390/plants13172410 PMC11397454 39273894

[24] S. Donoso , K. Peña‐Rojas , S. Durán , C. Pacheco , E. Galdames , and C. Espinoza , “Influencia del Raleo en el Crecimiento, la Condición Hídrica y la Respuesta Fotosintética de *Peumus boldus*: Pautas para la Definición de Su Manejo Silvicultural,” Bosque (Valdivia) 36 (2015): 457–466, 10.4067/S0717-92002015000300012.

[25] R. Gangas , S. Donoso , C. Espinoza , et al., “Evaluación en Árboles De *Peumus boldus* Después de 10 Años Desde la Cosecha: Respuesta en un Escenario de Megasequía,” Bosque (Valdivia) 44 (2023): 535–546, 10.4067/s0717-92002023000300535.

[26] S. Benedetti , F. Balocchi , and M. Hormazábal , “Arbuscular Mycorrhizal Fungi (AMF) Linked to *Peumus boldus* Natural Formation in Central Chile,” Gayana Botanica 75 (2018): 431–437, 10.4067/S0717-66432018000100431.

[27] S. A. R. Atala and M. A. Molina‐Montenegro , “Assessing the Importance of Native Mycorrhizal Fungi to Improve Tree Establishment after Wildfires,” Journal of Fungi 9 (2023): 421, 10.3390/jof9040421. PMC10144394 37108876

[28] R. Santelices‐Moya , A. Cabrera‐Ariza , F. Ávila‐Rojas , and P. Silva‐Flores , “Removal of the Epi‐ and Mesocarp from the Semi‐Mature Fruit of *Peumus boldus* and Not the Use of Gibberellic Acid Promotes the Highest Seed Germination,” International Journal of Agriculture and Natural Resources 47 (2020): 145–151, 10.7764/ijanr.v47i2.2203.

[29] R. Magni , S. E. Espinoza , E. F. Garrido , et al., “Differential Adaptations in Nursery Seedlings From Diverse Chilean Provenances of *Peumus boldus* Mol,” iForest—Biogeosciences and Forestry 9 (2016): 409–413, 10.3832/ifor1893-008.

[30] P. Bickford , “Ecophysiology of Leaf Trichomes,” Functional Plant Biology 43 (2016): 807–814, 10.1071/FP16095. 32480505

[31] C. C. C. Teixeira , T. P. de Freitas Cabral , J. P. B. de Sousa , S. de Pádua Teixeira , J. K. Bastos , and L. A. P. de Freitas , “Study of Quality Assurance for *Peumus boldus* M. Products by Botanic Profiling, Extraction Optimization, HPLC Quantification and Antioxidant Assay,” Pharmacognosy Journal 8 (2016): 264–272.

[32] B. Argüelles‐Marrón , J. A. Meave , I. Luna‐Vega , et al., “Adaptation Potential of Neotropical Montane Oaks to Drought Events: Wood Anatomy Sensitivity in *Quercus delgadoana* and *Quercus meavei* ,” Functional Ecology 37 (2023): 2040–2055, 10.1111/1365-2435.14362.

[33] B. K. Cassels , G. Fuentes‐Barros , and S. Castro‐Saavedra , “Boldo, Its Secondary Metabolites and Their Derivatives,” Current Traditional Medicine 5 (2019): 31–65, 10.2174/2215083804666181113112928.

[34] J. M. Prado and M. A. Rostagno , eds., Natural Product Extraction: Principles and Applications (Royal Society of Chemistry, 2022).

[35] M. Lück , F. Bergs , and A. Jupke , “Solvent Accessibility Limitation by Plant Matrix Compounds in Extraction of Rutin From *Solanum lycopersicum* ,” Separation Science Plus 3 (2020): 63–71.

[36] L. M. Anaya‐Esparza , E. F. Aurora‐Vigo , Z. Villagrán , et al., “Design of Experiments for Optimizing Ultrasound‐Assisted Extraction of Bioactive Compounds From Plant‐Based Sources,” Molecules 28 (2023): 7752, 10.3390/molecules28237752. PMC10707804 38067479

[37] H. K. Kala , R. Mehta , K. K. Sen , et al., “Critical Analysis of Research Trends and Issues in Microwave Assisted Extraction of Phenolics: Have We Really Done Enough,” TrAC Trends in Analytical Chemistry 85 (2016): 140–152, 10.1016/j.trac.2016.09.007.

[38] J. M. del Valle , T. Rogalinski , C. Zetzl , and G. Brunner , “Extraction of *Peumus boldus* M. Leaves With Supercritical CO_2_ and Hot Pressurized Water,” Food Research International 38 (2005): 203–213.

[39] P. A. Uwineza and A. Waśkiewicz , “Recent Advances in Supercritical Fluid Extraction of Natural Bioactive Compounds From Natural Plant Materials,” Molecules 25 (2020): 3847, 10.3390/molecules25173847. PMC7504334 32847101

[40] C. V. Madrid , V. Olave , et al., “Anti‐yeast Activity of the Essential Oil of Peumus Boldus Mol. From the Coastal Drylands of Central Chile Against Strains That Cause Uropharyngeal and Vulvovaginal Candidiasis,” Boletin Latinoamericano y del Caribe de Plantas Medicinales y Aromaticas 24 (2025): 298–307, 10.37360/blacpma.25.24.2.22.

[41] S. N. Lopes , I. R. Guesdon , G. M. Corrêa , et al., “Chemical Composition and Biological Activities of the Essential Oil of *Peumus boldus* Molina (Monimiaceae),” Revista Virtual de Química 12 (2020): 433–446, 10.21577/1984-6835.20200035.

[42] J. H. Bakiri and R. Reynaud , “Computer‐Aided ^13^C NMR Chemical Profiling of Crude Natural Extracts Without Fractionation,” Journal of Natural Products 80 (2017): 1387–1396, 10.1021/acs.jnatprod.6b01063. 28414230

[43] A. C. Ferrante , P. Angelini , R. Venanzoni , et al., “Phenolic Content and Antimicrobial and Anti‐Inflammatory Effects of *Solidago virga‐aurea*, *Phyllanthus niruri*, *Epilobium angustifolium*, *Peumus boldus*, and *Ononis spinosa* Extracts,” Antibiotics 9 (2020): 783, 10.3390/antibiotics9110783. PMC7694769 33172081

[44] N. Keivani , V. Piccolo , A. Marzocchi , et al., “Optimization and Validation of Procyanidins Extraction and Phytochemical Profiling of Seven Herbal Matrices of Nutraceutical Interest,” Antioxidants 13 (2024): 586, 10.3390/antiox13050586. PMC11117784 38790691

[45] Y. C.‐N. de Jesús Terán‐Sánchez , E. A. Herrera‐Jurado , M. M. Montenegro‐Sustaita , E. Becerra‐Martínez , and L. G. Zepeda‐Vallejo , “ ^1^H NMR‐Based Chemical Profiling of Retail Samples of *Peumus boldus* (Boldo) Leaves,” Journal of the Mexican Chemical Society 69 (2025): 621–635.

[46] M. C. Bianchini , C. O. A. Gularte , D. F. Escoto , et al., “ *Peumus boldus* (Boldo) Aqueous Extract Present Better Protective Effect Than Boldine Against Manganese‐Induced Toxicity in *D. melanogaster* ,” Neurochemical Research 41 (2016): 2699–2707, 10.1007/s11064-016-1984-z. 27350578

[47] R. Carmona , M. Reyes‐Díaz , J. Parodi , and C. Inostroza‐Blancheteau , “Antimutagenic Evaluation of Traditional Medicinal Plants From South America *Peumus Boldus* and *Cryptocarya alba* Using *Drosophila melanogaster* ,” Journal of Toxicology and Environmental Health, Part A 80 (2017): 208–217, 10.1080/15287394.2017.1279574. 28304234

[48] Y. K. da Silva , A. D. S. N. Lopes , P. J. S. Maia , et al., “Chemical and Biological Evaluation of the Aqueous Extract of *Peumus boldus* Molina (Monimiaceae) Leaves,” Pharmacognosy Research 14 (2022): 45–52.

[49] L. M. Vieitez , I. Jachmanián , and S. Alborés , “Antioxidant and Antibacterial Activity of Different Extracts From Herbs Obtained by Maceration or Supercritical Technology,” Journal of Supercritical Fluids 133 (2018): 58–64, 10.1016/j.supflu.2017.09.025.

[50] K. A. Santoro , R. Vesny , C. Navarro , H. Gatto , and R. Marsella , “Evaluation of the *in Vitro* Effect of Boldo and Meadowsweet Plant Extracts on the Expression of Antimicrobial Peptides and Inflammatory Markers in Canine Keratinocytes,” Research in Veterinary Science 115 (2017): 255–262, 10.1016/j.rvsc.2017.05.021. 28549300

[51] M. B. Santoro , K. Ahrens , C. Navarro , H. Gatto , and R. Marsella , “Evaluation of the Effects of 0.1% *Peumus boldus* Leaf and *Spiraea ulmaria* Plant Extract Combination on Bacterial Colonization in Canine Atopic Dermatitis: A Preliminary Randomized, Placebo‐Controlled, Double‐Blinded Study,” Research in Veterinary Science 118 (2018): 164–170, 10.1016/j.rvsc.2018.02.006. 29482177

[52] E. R. da Cruz , J. F. da Costa Guerra , M. de Souza Gomes , G. R. O. Freitas , and E. R. Morais , “Phytochemical Analysis and Evaluation of Antimicrobial Activity of *Peumus boldus*, *Psidium guajava*, *Vernonia polysphaera*, *Persea americana*, *Eucalyptus citriodora* Leaf Extracts and *Jatropha multifida* Raw Sap,” Current Pharmaceutical Biotechnology 20 (2019): 433–444. 10.2174/1389201020666190409104910 30963968

[53] L. B. M. Montaldo , M. De Marzi , and L. N. Guerra , “ *Peumus Boldus* Extract Inhibits Lipid Accumulation in 3T3‐L1 Adipocytes,” International Journal of Molecular Sciences 26 (2025): 4326, 10.3390/ijms26094326. PMC12072887 40362563

[54] N. S. Girardi , D. García , S. N. Robledo , et al., “Microencapsulation of *Peumus boldus* Oil by Complex Coacervation to Provide Peanut Seeds Protection against Fungal Pathogens,” Industrial Crops and Products 92 (2016): 93–101, 10.1016/j.indcrop.2016.07.045.

[55] N. S. Girardi , M. A. Passone , D. Garcia , et al., “Essential Oil Microencapsulation Technology to Provide Peanut Seeds Protection against Fungal Pathogens,” in Agricultural Research Updates, Vol. 21 (Nova Science Publishers, 2017), 213–242.

[56] N. S. Girardi , M. A. Passone , D. García , et al., “Microencapsulation of *Peumus boldus* Essential Oil and Its Impact on Peanut Seed Quality Preservation,” Industrial Crops and Products 114 (2018): 108–114, 10.1016/j.indcrop.2018.01.036.

[57] M. M. Abo Ghanima , M. Bin‐Jumah , A. M. E. Abdel‐Moneim , et al., “Impacts of Strain Variation on Response to Heat Stress and Boldo Extract Supplementation to Broiler Chickens,” Animals 10 (2020): 24. 10.3390/ani10010024 PMC7023343 31877662

[58] M. Platzer , S. Kiese , T. Herfellner , U. Schweiggert‐Weisz , O. Miesbauer , and P. Eisner , “Common Trends and Differences in Antioxidant Activity Analysis of Phenolic Substances Using Single Electron Transfer‐Based Assays,” Molecules 26 (2021): 1244, 10.3390/molecules26051244. PMC7956415 33669139

[59] S. M. Ahmed , L. Luo , A. Namani , X. J. Wang , and X. Tang , “Nrf2 Signaling Pathway: Pivotal Roles in Inflammation,” Biochimica et Biophysica Acta (BBA)—Molecular Basis of Disease 1863 (2017): 585–597, 10.1016/j.bbadis.2016.11.005. 27825853

[60] T. Liu , L. Zhang , D. Joo , and S. C. Sun , “NF‐κB Signaling in Inflammation,” Signal Transduction and Targeted Therapy 2 (2017): 17023, 10.1038/sigtrans.2017.23. PMC5661633 29158945

[61] D. Lamba , D. K. Dwivedi , M. Yadav , and Y. S. Kumar , “Boldine: A Narrative Review of the Bioactive Compound with Versatile Biological and Pharmacological Potential,” Journal of Complementary and Integrative Medicine 21 (2024): 269–281, 10.1515/jcim-2023-0224. 38234264

[62] K. Cassels , S. Castro‐Saavedra , and G. Fuentes‐Barros , “Chapter 21: Boldine,” in A Centum of Valuable Plant Bioactives (Academic Press, 2021), 491–508, 10.1016/B978-0-12-822923-1.00006-6.

[63] T. S. Hostalkova and J. Chlebek , “Boldine Alkaloids and Prospects of Their Utilization,” Chemické Listy 109 (2015): 846–855.

[64] M. B. G. de Almeida Figueiredo , V. R. de Santana , M. J. Nardelli , et al., “The Effect of the Aqueous Extract *Peumus boldus* on the Proliferation of Hepatocytes and Liver Function in Rats Submitted to Expanded Hepatectomy,” Acta Cirurgica Brasileira 31 (2016): 608–614. 10.1590/S0102-865020160090000006 27737346

[65] A. B. S. Refaie and H. F. Booles , “Hepatoprotective Impact of Boldo (*Peumus boldus*) Extract against Azoxystrobin‐Induced DNA Damage, Gene Expression Modulation, Biochemical and Histopathological Alterations Mediated by ROS Generation in Male Rats,” Egyptian Journal of Chemistry 65 (2022): 687–698.

[66] G. Rosales Muñoz , C. Soria Fregozo , and M. I. Pérez Vega , “Efecto Hepatoprotector de Una Mezcla de Siete Plantas en Cirrosis Inducida con Tetracloruro de Carbono,” Revista Cubana de Plantas Medicinales 22 (2017): e0.

[67] A. C. Orlando and C. Ferrante , “Protective Effects Induced by the Food Supplement Fluxonorm in the Lower Urinary Tract,” European Review for Medical and Pharmacological Sciences 25 (2021): 3074–3082. 10.26355/eurrev_202104_25562 33877671

[68] V. Polanco , D. Cerdá‐Bernad , I. Quispe‐Fuentes , C. Bernal , and J. López , “Bioactive Content and Antioxidant Properties of Spray‐Dried Microencapsulates of *Peumus boldus* M. Leaf Extracts,” Antioxidants 13 (2024): 1568, 10.3390/antiox13121568. PMC11673136 39765896

[69] W. Bertan , G. L. Aparecida , J. Bonilla , et al., “Boldo (*Peumus boldus*) Leaf's Hydroethanolic Extracts on Gelatin‐Based Active Films,” Journal of Food Processing and Preservation 45 (2021): e15936, 10.1111/jfpp.15936.

[70] S. Bhambhani , K. R. Kondhare , and A. P. Giri , “Diversity in Chemical Structures and Biological Properties of Plant Alkaloids,” Molecules 26 (2021): 3374, 10.3390/molecules26113374. PMC8199754 34204857

[71] Y. Tian , Z. An , Q. Li , et al., “Biosynthesis of Benzylisoquinoline Alkaloids and Its Evolution in Plants,” Plant Communications 7 (2026): 101786, 10.1016/j.xplc.2026.101786. PMC12983256 41776991

[72] B. K. Cassels , M. Asencio , P. Conget , et al., “Structure–Antioxidative Activity Relationships in Benzylisoquinoline Alkaloids,” Pharmacological Research 31 (1995): 103–107, 10.1016/1043-6618(95)80054-9. 7596952

[73] B. K. C. Speisky , E. A. Lissi , and L. A. Videla , “Antioxidant Properties of the Alkaloid Boldine in Systems Undergoing Lipid Peroxidation and Enzyme Inactivation,” Biochemical Pharmacology 41 (1991): 1575–1581, 10.1016/0006-2952(91)90156-Y. 2043147

[74] K. Kim , S. J. Kim , W. J. Gil , and C. S. Yang , “Exploring the Therapeutic Potential of Phytochemicals: Challenges and Strategies for Clinical Translation,” Phytomedicine 145 (2025): 157090, 10.1016/j.phymed.2025.157090. 40716124

[75] A. B. de Souza , V. M. Oliveira , L. S. Soares , N. R. Baldoni , F. M. D. Chequer , and R. O. do Couto , “Safety of Boldo Phytomedicines Following Systemic Administration: A Systematic Review of Pre‐Clinical Studies,” Revista Brasileira de Plantas Medicinais 25 (2023): 29–44.

[76] C. D. Lorenzo , A. Ceschi , H. Kupferschmidt , et al., “Adverse Effects of Plant Food Supplements and Botanical Preparations: A Systematic Review with Critical Evaluation of Causality,” British Journal of Clinical Pharmacology 79 (2015): 578–592, 10.1111/bcp.12519. PMC4386943 25251944

[77] A. O. Sá , T. Pimentel , and N. Oliveira , “Boldo‐Induced Hepatotoxicity: A Case of Unexplained Jaundice,” European Journal of Case Reports in Internal Medicine 7 (2020): 002116, 10.12890/2020_002116. PMC7806290 33457373

[78] R. J. Andrade , I. Medina‐Caliz , A. Gonzalez‐Jimenez , M. Garcia‐Cortes , and M. I. Lucena , “Hepatic Damage by Natural Remedies,” Seminars in Liver Disease 38 (2018): 21–40. 10.1055/s-0038-1623518 29471563

[79] P. A. Borges , B. B. D. Silva , R. M. Fernandes , et al., “Hepatotoxic Medicinal Plants in Weight Loss Formulations Sold at Ver‐o‐Peso Market, Amazon Region, Brazil,” Journal of Medicinal Food 28 (2025): 212–217, 10.1089/jmf.2024.0188. 39514333

[80] Ó. B. Portugal and M. Flores‐Quispe , “Concentrations of Arsenic, Cadmium, and Lead in Herbal Infusion Tea Bags Marketed in Tacna, Peru,” Environmental Monitoring and Assessment 194 (2022): 534, 10.1007/s10661-022-10232-3. 35764709

[81] European Medicines Agency, Committee on Herbal Medicinal Products (HMPC), European Union Herbal Monograph on *Peumus boldus* Molina, *Folium*, EMA/HMPC/453725/2016 (2016).

[82] R. Pavela , G. Benelli , R. Petrelli , et al., “Exploring the Insecticidal Potential of Boldo (*Peumus boldus*) Essential Oil: Toxicity to Pests and Vectors and Non‐Target Impact on the Microcrustacean *Daphnia magna* ,” Molecules 24 (2019): 879, 10.3390/molecules24050879. PMC6429277 30832296

[83] J.‐P. Lambert and J. Cormier , “Potential Interaction between Warfarin and Boldo‐Fenugreek,” Pharmacotherapy 21 (2001): 509–512, 10.1592/phco.21.5.509.34492. 11310527

[84] R. Carbajal , A. Yisfalem , N. Pradhan , D. Baumstein , and A. Chaudhari , “Case Report: Boldo (*Peumus boldus*) and Tacrolimus Interaction in a Renal Transplant Patient,” Transplantation Proceedings 46 (2014): 2400–2402, 10.1016/j.transproceed.2014.01.021. 24981811

[85] S. Nawaz , H. M. Irfan , Alamgeer , et al., “Attenuation of CFA‐Induced Chronic Inflammation by a Bicyclic Monoterpene Fenchone Targeting Inducible Nitric Oxide, Prostaglandins, C‐Reactive Protein and Urea,” Inflammopharmacology 31 (2023): 2479–2491, 10.1007/s10787-023-01333-7. 37689616

[86] E. C. Araruna , E. B. A. Júnior , C. A. D. L. Serafim , et al., “(‐)‐Fenchone Prevents Cysteamine‐Induced Duodenal Ulcers and Accelerates Healing Promoting Re‐Epithelialization of Gastric Ulcers in Rats via Antioxidant and Immunomodulatory Mechanisms,” Pharmaceuticals 17 (2024): 641, 10.3390/ph17050641. PMC11124074 38794211

[87] L. S. Pessoa , L. M. O. Silva , M. E. C. Araruna , et al., “Antifungal Activity and Antidiarrheal Activity via Antimotility Mechanisms of (‐)‐Fenchone in Experimental Models,” World Journal of Gastroenterology 26 (2020): 6795–6809, 10.3748/wjg.v26.i43.6795. PMC7684460 33268962

[88] M. N. M. Bashir , W. Younis , and I. Anjum , “Fenchone, a Monoterpene: Toxicity and Diuretic Profiling in Rats,” Frontiers in Pharmacology 14 (2023): 1119360, 10.3389/fphar.2023.1119360. PMC9909529 36778012

[89] T. Boeing , L. N. B. Mariano , and A. C. Dos Santos , “Gastroprotective Effect of the Alkaloid Boldine: Involvement of Non‐Protein Sulfhydryl Groups, Prostanoids and Reduction of Oxidative Stress,” Chemico‐Biological Interactions 327 (2020): 109166, 10.1016/j.cbi.2020.109166. 32531310

[90] K. Chen , Z. T. Lv , P. Cheng , et al., “Boldine Ameliorates Estrogen Deficiency‐Induced Bone Loss via Inhibiting Bone Resorption,” Frontiers in Pharmacology 9 (2018): 1046, 10.3389/fphar.2018.01046. PMC6146032 30271347

[91] P. Toledo , E. J. Fernández‐Pérez , I. L. Ferreira , et al., “Boldine Attenuates Synaptic Failure and Mitochondrial Deregulation in Cellular Models of Alzheimer's Disease,” Frontiers in Neuroscience 15 (2021): 617821, 10.3389/fnins.2021.617821. PMC7933475 33679301

[92] C. L. Silva , G. H. de Souza , V. O. Pateis , et al., “Inhibition of Gluconeogenesis by Boldine in the Perfused Liver: Therapeutical Implication for Glycemic Control,” International Journal of Hepatology 2023 (2023): 1283716, 10.1155/2023/1283716. PMC10089784 37056327

[93] H. Turgut , H. Gungor , M. Ekici , M. A. Erdogan , M. O. Karayigit , and H. Kara , “Boldine Provides Protective Effect against Nephrotoxicity Induced by Cisplatin in Wistar Rats: Role of Oxidative Stress, Inflammation and Caspase‐3,” Biocell 46 (2022): 2111–2122, 10.32604/biocell.2022.020383.

[94] I. Gómez and V. Velarde , “Boldine Improves Kidney Damage in the Goldblatt 2K1C Model Avoiding the Increase in TGF‐β,” International Journal of Molecular Sciences 19 (2018): 1864. 10.3390/ijms19071864 PMC6073111 29941815

[95] V. Calbiague‐García , B. Cadiz , P. Herrera , et al., “Evaluation of Photobiomodulation and Boldine as Alternative Treatment Options in Two Diabetic Retinopathy Models,” International Journal of Molecular Sciences 24 (2023): 7918, 10.3390/ijms24097918. PMC10178531 37175628

[96] M. P. Mondal , A. K. Panigrahi , and A. R. Khuda‐Bukhsh , “Improved Drug Carriage and Protective Potential against Cisplatin‐Induced Toxicity Using Boldine‐Loaded PLGA Nanoparticles,” Journal of Ayurveda and Integrative Medicine 11 (2020): 24–36, 10.1016/j.jaim.2017.11.002. PMC7125378 30115410

[97] M. F. Shuker , G. Al‐Qudaihi , and D. Fouad , “Potential Effects of Boldine on Oxidative Stress, Apoptosis, and Inflammatory Changes Induced by the Methylprednisolone Hepatotoxicity in Male Wistar Rats,” Dose‐Response 20 (2022): 15593258221079077, 10.1177/15593258221082877. PMC8961387 35360456

[98] P. Subramaniam , A. K. Kannan , and D. Thiruvengadam , “Hepatoprotective Effect of Boldine against Diethylnitrosamine‐Induced Hepatocarcinogenesis in Wistar Rats,” Journal of Biochemical and Molecular Toxicology 33 (2019): e22404, 10.1002/jbt.22404. 31593341

[99] P. K. Subramaniam , J. Sundaram , A. Mari , S. K. Velli , and S. Salam , “Potential Chemopreventive Role of Boldine Against Hepatocellular Carcinoma via Modulation of Cell Cycle Proteins in Rat Model,” Anti‐Cancer Agents in Medicinal Chemistry 21 (2021): 2546–2552, 10.2174/1871520621666210203102854. 33535961

[100] A. S. Caione and G. Collura , “Phytotherapy as Ancillary Treatment after Urinary Stone Lithotripsy in Pediatric Age,” Annali Italiani di Chirurgia 93 (2022): 313–318. 36052473

